# Reconciling Binary Replicates: Beyond the Average

**DOI:** 10.1002/sim.70416

**Published:** 2026-02-05

**Authors:** H. Lorenzo, P. Pudlo, M. Royer‐Carenzi

**Affiliations:** ^1^ UMR 7373, CNRS, Centrale Marseille, I2M Aix Marseille Univ Marseille France

**Keywords:** Bayesian algorithm, expectation–maximization algorithm, median, medical diagnosis, prevalence estimation, technical replicates

## Abstract

Binary observations are often repeated to improve data quality, creating technical replicates. Several scoring methods are commonly used to infer the actual individual state and obtain a probability for each state. The common practice of averaging replicates has limitations, and alternative methods for scoring and classifying individuals are proposed. Additionally, an indecisive response might be wiser than classifying all individuals based on their replicates in the medical context, where 1 indicates a particular health condition. Building on the inherent limitations of the averaging approach, three alternative methods are examined: the median, maximum penalized likelihood estimation, and a Bayesian algorithm. The theoretical analysis suggests that the proposed alternatives outperform the averaging approach, especially the Bayesian method, which incorporates uncertainty and provides credible intervals. Simulations and real‐world medical datasets are used to demonstrate the practical implications of these methods for improving diagnostic accuracy and disease prevalence estimation.

## 1 Introduction

In Medicine, Biology, and, more generally, applied sciences, conclusions are drawn from carefully analyzing noisy data. Noise may come from the measurement protocol, the instruments, or the intrinsic variability of the phenomenon under study. In this context, using technical replicates is a common practice to improve the robustness of the conclusions. Approaches based on replication groups have been studied to reduce the false positive rate in replicate analysis [1]. As Palmer said [2] “Measurement errors are unavoidable, and repetitions should be the rule to quantify its magnitude.” However, dealing with technical replicates is not always straightforward: replicates of the same individuals are more likely to be similar than replicates of different individuals. This phenomenon is known as overdispersion, and it has been widely studied in the literature [3, 4, 5, 6]. Jaeger [4] showed that not taking into account the correlation of the responses for the same subject leads to biased estimates and artificially increases our trust in the estimators, which can lead to invalid statistical analyses. A common practice to handle this correlation is to summarize the replicates of the same individual by a single score, which is usually the empirical average of the replicates. The distinction between technical and biological replicates is essential to avoid misinterpretation in medical studies [7, 8]. This paper focuses on binary data: each replicate is a binary variable, and the individual's actual state is also a binary variable. The leading difficulty is that a binary variable carries very little information, and noise can change its value to its opposite. In medical diagnostics, the actual state equals 1 when the subject has the disease and 0 otherwise. The replicates are binary values that aim to infer the individual's actual state.

Dealing with binary replicates in medical studies involves addressing the challenges of repeated binary outcomes, which are common in longitudinal studies and clinical trials [9, 10, 11, 12]. These outcomes are often correlated, necessitating specialized statistical methods for accurate analysis. The use of correlated binomial models has been proposed to take into account the dependence between technical replicates, enabling better estimation of error rates [13]. We need at least to account for within‐patient correlation in these data. Various approaches have been developed to handle these complexities, each with advantages and limitations. The recent literature dates back to 1982 [3]. In 1990, Hujoel et al. [6] introduced a correlated binomial model that can be employed to estimate the sensitivity and specificity of these diagnostic tests [14, 15]. Various approaches have been developed to construct robust confidence intervals in the presence of correlated binary data [16]. Contemporary methods emphasize integrating advanced biomarkers and machine learning algorithms for improved diagnostic performance. Yet there has been recent interest in improving the results on repeated binary data, with modern methods such as the Bayesian machinery of a latent class model [17]. Two‐phase sampling has been used to improve the efficiency of parameter estimation in longitudinal binary data contexts [18]. However, few theoretical results have been published on the accuracy of these methods. This paper intends to fill in this gap in a relatively simple case.

We introduce and compare four statistical methods: an average‐based, a median‐based, a maximum‐a‐posteriori (MAP)‐based, and a Bayesian method. Each method provides a different way of scoring the individuals. We design a classifier for each approach that makes decisions for each individual and returns a trustworthy diagnostic. We propose different methods to estimate the prevalence of the disease in the sampled population and the sensitivity and specificity of the binary replicate without resorting to a gold standard test. With a relatively simple framework, we intend to provide theoretical results comparing the proposed methods illustrated in various numerical cases. Moreover, we introduce an indecision outcome in the individuals' classification, which is returned in cases of significant doubt. This helps us understand better the information provided by the multiple scorings of the individuals and better fit real‐world medical instances in which erroneous decisions can have serious consequences. In addition, we examine the robustness of the proposed methods under two challenging scenarios: first, when most individuals have only one or two measurements, and second, when error rates vary across individuals. These real‐world situations provide an opportunity to evaluate whether the methods maintain their effectiveness outside the ideal modeling framework.

We organized the paper as follows. Section 2 describes the statistical model, the four competing approaches, and their rationale. Section 3 gives mathematical results to compare their efficiency in terms of both classification and estimation. In Section 4, simulations are used to compare the methods, and numerical illustrations on real‐world medical datasets are provided: a periodontal dataset [6] and a mammogram screening dataset [19, 20]. The methods described in the paper are implemented in an R‐package, available on GitHub at the following address: https://github.com/pierrepudlo/BinaryReplicates.

## 2 Methods

In Section 2.1, we introduce a statistical model for binary replicates that intends to capture the binary state of an individual. To infer the prevalence of each state in the population and to classify the individuals, we introduce four statistical methods: an average‐based, a median‐based, a MAP‐based, and a Bayesian method. All these methods rely on scoring the individuals given their binary replicates; see Sections 2.3, 2.5. Finally, for each method, we introduce classifiers to recover the binary state of each individual based on the scoring and estimators of the prevalence in Section 2.7.

### 2.1 The Statistical Model

We consider a dataset comprising binary observations $X_{\mathrm{ij}}\in 0,1$, where i=1,…,N indexes individuals and $j=1,\ldots ,n_{i}$ indexes technical replicates. Each replicate $X_{\mathrm{ij}}$ approximates the true status $T_{i}\in 0,1$, used to mitigate measurement imprecision. For instance, in medical diagnostics, $T_{i}=1$ denotes disease presence, while $T_{i}=0$ indicates health. We assume the replicates $X_{\mathrm{ij}}$ are independent noisy measurements of $T_{i}$. The marginal distribution of $T_{i}$ is Bernoulli with parameter $\theta_{T}$, representing disease prevalence. For each replicate j,

$$\begin{array}{l} p=P\;\left(X_{\mathrm{ij}}=1|T_{i}=0\right)\;\text{is the false}‐\text{positivity rate},\\ q=P\;\left(X_{\mathrm{ij}}=0|T_{i}=1\right)\;\text{is the false}‐\text{negativity rate}. \end{array}$$

The measurement error $T_{i}-X_{\mathrm{ij}}$ takes values in −1, 0, 1, with 0 indicating no error, 1 a false positive, and −1 a false negative.

By the law of total probability, $E\left(T_{i}-X_{\mathrm{ij}}\right)=\theta_{T}q+\left(\theta_{T}-1\right)p$, which is non‐zero if $\theta_{T}\neq p/\left(p+q\right)$. Thus, the diagnostic test is biased under these conditions. If either p or q exceeds 1/2, the replicate is more likely wrong than correct, resulting in unreliable data. In what follows, we assume that p,q∈(0,1/2).

### 2.2 Sufficient Statistics

We summarize the entire dataset of $X_{\mathrm{ij}}$, for i=1,…,N and $j=1,\ldots ,n_{i}$, by introducing the sum $S_{i}=X_{i1}+\cdots +X_{{\text{in}}_{i}}$ for each individual i. This sum represents the number of positive replicates for individual i. The vector $\left(S_{1},\ldots ,S_{N}\right)$ is a sufficient statistic for the dataset. Indeed, the only information lost in this transformation is the order of the replicates for each individual. However, since the technical replicates for a given individual are exchangeable, their order carries no relevance to the statistical analysis. Therefore, no statistical information is lost when we replace the dataset with the vector $\left(S_{1},\ldots ,S_{N}\right)$.

Given $T_{i}=1$, respectively $T_{i}=0$, the technical replicates $X_{\mathrm{ij}}$ are independent Bernoulli random variables with parameter 1−q, respectively p. Thus, $S_{i}$ given the true value $T_{i}$ is as follows:

(1) $$\begin{array}{ll} & \left[S_{i}|T_{i}\right]∼B\;\text{in}\left(n_{i},T_{i}\left(1-q\right)+\left(1-T_{i}\right)p\right)\\ & \;=1\;l_{T_{i}=1}B\;\text{in}\left(n_{i},1-q\right)+1\;l_{T_{i}=0}B\;\text{in}\left(n_{i},p\right), \end{array}$$

with independence between the $S_{i}$'s given $\left(T_{1},\ldots ,T_{N}\right)$.

### 2.3 Scoring Technical Replicates With the Mean and the Median

The average‐based score $Y_{A,i}$ is defined as follows:

$$Y_{A,i}=\frac{1}{n_{i}}\sum_{j=1}^{n_{i}}X_{\mathrm{ij}}=\frac{S_{i}}{n_{i}}.$$

This score is widely used, as it is a simple scaling of the sufficient statistic $S_{i}$.

The median‐based score $Y_{M,i}$ is defined as follows:

(2) $$Y_{M,i}=\text{median}\left(X_{i1},\ldots ,X_{{\text{in}}_{i}}\right)=1\;l_{S_{i}>n_{i}/2}+\frac{1}{2}1\;l_{S_{i}=n_{i}/2}.$$

In the specific case where $n_{i}$ is even and when there is a tie in the frequencies of 0 and 1 within the replicates of individual i, we set $Y_{M,i}=1/2$.

Note that with the above definitions, the mean and median scores are equal when $n_{i}\in \left\{1,2\right\}$. However, their effectiveness is not guaranteed, and we will compare them with other scoring methods that are non‐linear functions of $S_{i}$.

### 2.4 MAP Scoring

The most likely value of $T_{i}$ given the observed values of $S_{i}$ depends on the value of $P\;\left(T_{i}=1|S_{i}=s_{i}\right)$, whether below or above 1/2. Using (1), $T_{i}∼B\;\mathrm{er}\left(\theta_{T}\right)$ and the Bayes formula, the distribution of $T_{i}$ given $S_{i}$ is as follows:

(3) $$\left[T_{i}|S_{i}=s_{i}\right]∼B\;\mathrm{er}\left(\frac{\theta_{T}q^{n_{i}-s_{i}}{\left(1-q\right)}^{s_{i}}}{\theta_{T}q^{n_{i}-s_{i}}{\left(1-q\right)}^{s_{i}}+\left(1-\theta_{T}\right)p^{s_{i}}{\left(1-p\right)}^{n_{i}-s_{i}}}\right).$$

Thus, we introduce the likelihood‐based score $Y_{L,i}\left(\theta_{T},p,q\right)$ as follows

(4) $$\begin{array}{ll} & Y_{L,i}\left(\theta_{T},p,q\right)=P\;\left(T_{i}=1|S_{i}=s_{i}\right)\\ & \;=\frac{\theta_{T}q^{n_{i}-s_{i}}{\left(1-q\right)}^{s_{i}}}{\theta_{T}q^{n_{i}-s_{i}}{\left(1-q\right)}^{s_{i}}+\left(1-\theta_{T}\right)p^{s_{i}}{\left(1-p\right)}^{n_{i}-s_{i}}}. \end{array}$$

This score is an increasing function of $s_{i}$ because of Lemma 2 in Appendix A: On the Likelihood‐Based Scores. This score $Y_{L,i}\left(\theta_{T},p,q\right)$ depends on the values of p, q, and $\theta_{T}$. Estimating the fixed parameters $\theta_{T}$, p, and q by maximum likelihood is possible. Yet, we need to penalize the likelihood to avoid degenerate solutions. The likelihood of the fixed parameters $\theta_{T}$, p, and q given the data $\left(s_{1},\ldots ,s_{N}\right)$ is as follows:

(5) $$L\left(\theta_{T},p,q\right)=\prod_{i=1}^{n}\left\{\theta_{T}{\left(1-q\right)}^{s_{i}}q^{n_{i}-s_{i}}+\left(1-\theta_{T}\right)p^{s_{i}}{\left(1-p\right)}^{n_{i}-s_{i}}\right\},$$

up to a multiplicate constant. Besides, this maximum might suffer from unrealistic maxima, corresponding to p=0 or q=0, for example. For this reason, we penalize the likelihood with a Beta(2,2)‐prior on both p and q to avoid p=0 or q=0. The maximum of the corresponding functional, the MAP, is not explicit. As for maximum likelihood, we need to resort to numerical optimization. The standard algorithm to fulfill this task in mixture models is the Expectation–Maximization (EM) algorithm; see, for example, Chapters 1, 2, and 9 [21]. For further details, we refer to Appendix A: Full Description of the Maximum‐A‐Posteriori EM Estimation Algorithm.

By plugging in the result $\left({\hat{\theta }}_{T,M\;A\;P},{\hat{p}}_{M\;A\;P},{\hat{q}}_{M\;A\;P}\right)$ of the EM algorithm into (4), we define the MAP score $Y_{\mathrm{MAP},i}$ as follows:

(6) $$Y_{M\;A\;P,i}=Y_{L,i}\left({\hat{\theta }}_{T,M\;A\;P},{\hat{p}}_{M\;A\;P},{\hat{q}}_{M\;A\;P}\right).$$

### 2.5 Bayesian Scoring

**FIGURE 1:**
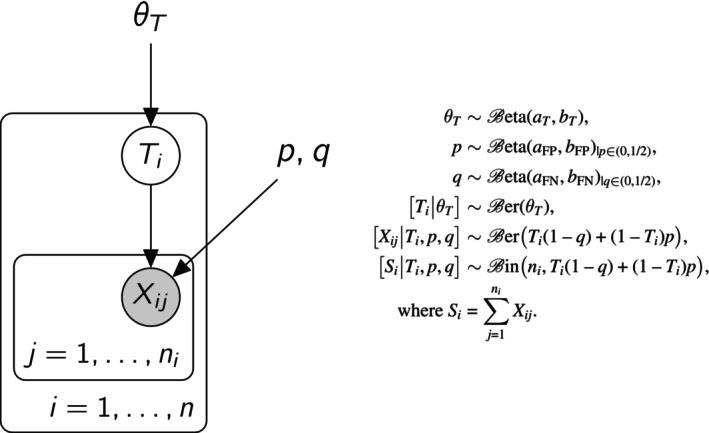
The Bayesian model: The Directed Acyclic Graph (left) and the generative model (right). Variables $\theta_{T}$, p, and q are fixed parameters (with a prior distribution), the $T_{i}$'s are latent variables, and the $X_{\mathrm{ij}}$'s are the observed data. The hyperparameters $a_{T},b_{T},a_{\mathrm{FP}},b_{\mathrm{FP}},a_{\mathrm{FN}},b_{\mathrm{FN}}$ set the prior distribution and should be chosen by the user. The prior distribution on p and q are Beta distributions truncated so that p and q are both in (0,1/2). The loop over the replicates $j=1,\ldots ,n_{i}$ can be replaced by a single node $S_{i}$, counting the number of ones among the $X_{\mathrm{ij}}$'s since it is a sufficient statistic.

We encompass the stochastic model described above in a Bayesian framework. We consider a Bayesian model with a prior distribution on $\theta_{T}$, p, and q whose density is denoted $\pi \left(\theta_{T},p,q\right)$, see Figure 1. In the Bayesian framework, $P_{\pi }$ and $E_{\pi }$ denote the probability measure and the expected value, as detailed in Appendix A: Bayesian Posterior Calculation.

Using the Bayesian model defined above and its posterior distribution, the Bayesian score $Y_{B,i}$ is defined as the following posterior expected value

$$Y_{B,i}=E_{\pi }\left(T_{i}|S_{1},\ldots ,S_{N}\right)=P_{\pi }\left(T_{i}=1|S_{1},\ldots ,S_{N}\right),$$

that integrates $T_{i}$ over the posterior values of $\theta_{T}$, p, and q and thus over the uncertainty on the fixed parameters. Alternatively, assuming $\pi \left(\theta_{T},p,q|S_{1},\ldots ,S_{N}\right)$ is the posterior distribution of the fixed parameters given the data, it can be viewed as the posterior expected value of the likelihood‐based score $Y_{L,i}\left(\theta_{T},p,q\right)=P\;\left(T_{i}=1|S_{i}\right)$, namely

(7) $$Y_{B,i}=\int Y_{L,i}\left(\theta_{T},p,q\right)\pi \left(\theta_{T},p,q|S_{1},\ldots ,S_{N}\right)d\theta_{T}dpdq.$$

Unlike scores $Y_{A,i}$ and $Y_{M,i}$, values of $Y_{M\;A\;P,i}$ and $Y_{B,i}$ depend not only on the replicates observed on the *i*th individual (i.e., $S_{i}$) but also on the whole dataset through the posterior distribution on the fixed parameters.

To decide how to set the hyperparameters for the prior distribution of the fixed parameters $\theta_{T}$, p, and q, see Figure 1, remember that each of these parameters represents the probability of getting a 1 in a binary (0/1) trial. For these probabilities, we use the Beta distribution Beta(a,b), where a,b>0. This distribution represents information from a sample of size a+b consisting of a observations equal to 1 and b equal to 0. A non‐informative prior is the Beta distribution with a=b=1/2, representing minimal prior knowledge. This can be thought of as a fictive sample of size 1 with equal likelihoods for 0 and 1. We recommend using this non‐informative prior for the prevalence $\theta_{T}$. The uniform distribution on (0,1) is a Beta distribution with a=b=1, providing a weakly informative prior based on a balanced sample of size 2. For the false‐positive rate p and the false‐negative rate q, setting $a_{\mathrm{FP}}=a_{\mathrm{FN}}=b_{\mathrm{FP}}=b_{\mathrm{FN}}=1/2$ is not ideal: this distribution places too much weight near 0, where the likelihood‐based scoring performs poorly. Since replicates imply noisy measurements, meaning p and q should not be too close to 0, we recommend using $a_{\mathrm{FP}}=a_{\mathrm{FN}}=b_{\mathrm{FP}}=b_{\mathrm{FN}}=2$, which puts less weight near 0. In summary, we propose using $a_{T}=b_{T}=1/2$ and $a_{\mathrm{FP}}=a_{\mathrm{FN}}=b_{\mathrm{FP}}=b_{\mathrm{FN}}=2$ as default values for the hyperparameters, adjusting them as needed if more information is available. See Figure 4, Section 4.2, for an example.

Finally, the median‐based score is a limit case of the Bayesian score. Specifically, when the prior is highly concentrated around $\left(\theta_{T},p,q\right)=\left(1/2,0,0\right)$, the posterior distribution of the fixed parameters also stays concentrated around (1/2,0,0). In this scenario, the Bayesian score $Y_{B,i}$ defined in (7) becomes approximately equal to $Y_{L,i}\left(1/2,0,0\right)$, which corresponds to the median‐based score $Y_{M,i}$. Thus, the median‐based score can be seen as an approximation of the Bayesian score when using a strongly informative prior. This strongly informative prior assumes that the false‐positive and false‐negative rates are negligible and that the prevalence is roughly 1/2. Such a situation occurs with the prior defined in Figure 1, when the hyperparameters $b_{\mathrm{FN}}=b_{\mathrm{FP}}\to \infty$ and $a_{T}=b_{T}\to \infty$ simultaneously.

### 2.6 From Scorings to Prevalence and Error Rates Estimators

To infer the prevalence $\theta_{T}$, we consider the four following estimates:

(8) $$\forall K\in \left\{A,M,M\;A\;P\right\},\;{\hat{\theta }}_{T,K}=\frac{1}{N}\sum_{i=1}^{N}Y_{K,i}\;\text{and}\;{\hat{\theta }}_{T,B}=E_{\pi }\left(\theta_{T}|S_{1},\ldots ,S_{N}\right).$$

For average‐, median‐ and MAP‐based methods, scorings can be used to estimate the false‐positivity and false‐negativity rates p and q:

(9) $$\forall K\in \left\{A,M,M\;A\;P\right\},\;{\hat{p}}_{K}=\frac{\sum_{i=1}^{N}S_{i}\left(1-Y_{K,i}\right)}{\sum_{i=1}^{N}n_{i}\left(1-Y_{K,i}\right)}\;\text{and}\;{\hat{q}}_{K}=\frac{\sum_{i=1}^{N}\left(n_{i}-S_{i}\right)Y_{K,i}}{\sum_{i=1}^{N}n_{i}Y_{K,i}}.$$

Bayesian statistics provides natural parameter estimates based on posterior expectations. We can approximate them easily by computing the average of a sample of $\theta_{T},p$ or q drawn from the posterior distribution with the Hamiltonian Monte Carlo algorithm of rstan.

### 2.7 From Scorings to Classifiers

We compute our predictions of the latent $T_{i}$ values by thresholding the scores. The above scoring statistics take values in [0,1] and summarize information from a given dataset, the *s*_*i*_'s, into a single value. We should interpret them as a tool to infer the latent $T_{i}$ value: when $T_{i}=1$, we expect high scores; when $T_{i}=0$, we expect low scores. We introduce an indecision response (1/2) when we would not trust a decision based on the observed replicates. Since binary data carry little information, this could happen. It is an invitation to add more replicates related to this individual before deciding.

To classify the individuals, we introduce two thresholds $0<v_{L}\leq 1/2\leq v_{U}<1$ and set the classifiers as follows:

(10) $${\hat{T}}_{K,i}=\Phi \left(Y_{K,i}\right),\;\text{where}\;\Phi \left(y\right)=\left\{\begin{array}{ll} 0 & \text{if}\;y<v_{L},\\ 1/2 & \text{if}\;v_{L}\leq y\leq v_{U},\\ 1 & \text{if}\;y>v_{U}, \end{array}\right.$$

for all methods K∈{A,M,MAP,B} and all individuals i=1,…,n. Since the median‐based score $Y_{M,i}$ is always in {0,1/2,1}, we always have ${\hat{T}}_{M,i}=Y_{M,i}$. This double thresholding method is related to risk theory. To compute the risk of a classifier, we introduce a loss function $\ell \left(t,\hat{t}\right)$ that quantifies the cost of predicting $\hat{t}\in \left\{0,1/2,1\right\}$ when the truth is t∈{0,1}. See Section 3.1 for more details.

### 2.8 Predictions

Let us assume that a new individual n+1 is given to the agent through $n_{n+1}$ and $s_{n+1}$. It is possible to give the posterior prediction of its score ${\hat{Y}}_{n+1}$ based on the dataset composed by the n previous individuals. For K∈{A,M,MAP}, we estimate the parameters ${\hat{\theta }}_{T,K},{\hat{p}}_{K}$ and ${\hat{q}}_{K}$, from the individuals {1,⋯,n}, and we compute

(11) $${\hat{Y}}_{K,n+1}=Y_{L,n+1}\left({\hat{\theta }}_{T,K},{\hat{p}}_{K},{\hat{q}}_{K}\right),$$

where $Y_{L,n+1}\left(\theta_{T},p,q\right)=P\;\left(T_{n+1}=1|S_{n+1}=s_{n+1}\right)$ is computed as in Equation (4). It is also possible to build the prediction score for the Bayesian approach, for which the form is

(12) $${\hat{Y}}_{B,n+1}=\int Y_{L,n+1}\left(\theta_{T},p,q\right)\;\pi \left(\theta_{T},p,q|S_{1},\ldots ,S_{n}\right)\;d\theta_{T}\;dp\;dq.$$

A Monte‐Carlo estimator is chosen to approximate the previous integral, such as follows:

$${\hat{Y}}_{B,n+1}=\frac{1}{H}\sum_{h=1}^{H}Y_{L,n+1}\left(\theta_{T,h},p_{h},q_{h}\right),$$

where each parameter $\left(\theta_{T,h},p_{h},q_{h}\right)$ is sampled from the posterior distribution $\pi \left(\theta_{T},p,q|S_{1},\ldots ,S_{n}\right)$ such as described in Section 2.5.

Finally, Bayesian‐prediction scores consider the uncertainty of the parameters estimates, contrary to other methods. Thus, non‐Bayesian prediction scores might lead to over‐confident decisions.

## 3 Theoretical Results

In this Section, we provide efficiency results that compare the various methods introduced above. We start with the efficiency of the classifiers in terms of sensitivity and specificity and of specific loss functions, as introduced in Section 3.1, that deal with indecision responses on the replicates. Section 3.2 gives the results on the classifiers. Section 3.3 gives the results on the prevalence estimators. To state the results, we may need the following hypotheses.

*The false‐positivity and false‐negativity rates* p *and* q *are in* (0,1/2).

*There exists at least one individual for which the number of replicates* $n_{i}\geq 3$.

*The* $v_{L}$ *and* $v_{U}$ *that define the classifiers in Section* 2.7 *are such that* $0<v_{L}\leq 1/2\leq v_{U}<1.$

*The loss function* $\ell \left(t,\hat{t}\right)$ *satisfies the Conditions (* *13* *) of Lemma* *1* *in Section* *3.1*.

### 3.1 Loss Functions for Classifiers With Indecision Response and Minimal Risk Classifiers

In this Subsection, we only look at the following problem. Assume we want to predict a binary random T∈{0,1}, with three possible decisions: 0, 1/2 (inconclusive) and 1 based on the known value of ϑ=P(T=1). (In the following section, ϑ can be $\theta_{T}$ or $P\;\left(T_{i}=1|S_{i}\right)$ if we reason given the data.)

Consider the loss function $\ell \left(t,\hat{t}\right)$, defined on {0,1}×{0,1/2,1} such as follows:

**Table:** 

$\ell \left(t,\hat{t}\right)$	0	1/2	1
0	0	a	b
1	c	d	0

where a, b, c and d are positive constants. When a=b=1 and c=d=0, the loss function is related to the specificity. When c=d=1 and a=b=0, the loss function is related to the sensitivity. And, when b=c=1 and a=d=0, the loss function is the misclassification error. The general loss function $\ell \left(t,\hat{t}\right)$ can be interpreted as follows. If a and d are small enough compared to b and c, the indecision response 1/2 may be the best choice in case of a substantial uncertainty between 0 and 1. In medical applications, asking for further tests may cost less than making a doubtful decision. An indecision response is always an error, whether the truth is 0 or 1. Therefore, decision methods propose $\hat{t}=1/2$ only if indecision costs are sufficiently low. The constraints are given in Equation (13) of Lemma 1. This gives a<1/2. However, this indecision cost a must remain high (i.e., not too close to 0) for decisions to be made in most cases.

Sometimes we would consider ℓ in symmetrical form, taking b=c=1 and a=d, denoted $\ell_{a}$, where 0<a<1 is the indecision cost. Thus the cost of a false positive, $\ell_{a}\left(0,1\right)$, is equal to the cost of a false negative, $\ell_{a}\left(1,0\right)$. This is unrealistic in the context of medical diagnosis. Indeed, we often wish to avoid false negatives not to leave a diseased patient untreated. In this case, an absence of decision with $\hat{t}=1/2$ is better than the false negative. The resulting cost of indecision, a, is thus lower than that of a false negative, set to 1.

For a given loss function ℓ, we are interested in the best classifiers ${\hat{T}}^{⋆}$ that minimize the risk $r\left(\hat{t}\right)=E\;\left(\ell \left(T,\hat{t}\right)\right)$, defined as follows:

$${\hat{T}}^{⋆}=\underset{\hat{t}\in \left\{0,1/2,1\right\}}{\arg\;\min}\;r\left(\hat{t}\right),$$

and we select the indecision response, that is, 1/2, if the risks tie in. Lemma 1 (a proof is given in Appendix A: Proof of Lemma 1) gives the conditions on a,b,c and d under which the indecision response can appear. It also provides the best decision in this case.

Lemma 1 *Assume* ϑ=P(T=1) *is known. The best classifier* ${\hat{T}}^{⋆}$ *is*

$${\hat{T}}^{⋆}=\Phi \left(\vartheta \right),$$

*where* Φ *is defined in Equation* (10) *with* $v_{L}=\frac{a}{c-\left(d-a\right)}$ *and* $v_{U}=\frac{b-a}{\left(d-a\right)+b}$

(13) $$\text{if}\;\text{and}\;\text{only}\;\text{if}\;\frac{\mathrm{bc}}{b+c}>a+\left(d-a\right)\frac{b}{b+c}\;\text{and}\;-b<\left(d-a\right)<c.$$

If we apply Lemma 1 to the symmetric loss function $\ell_{a}$, we must choose $v_{L}=a$ and $v_{U}=1-a$ to obtain the best classifier.

### 3.2 Accuracy of the Classifiers as a Diagnostic Tool

Whatever the scoring method, we rely on the same two thresholds $v_{L}\leq v_{U}$ to transform the scores into diagnostics. Let us denote

$$n_{0}=\max_{i}\;n_{i},\;\text{and}\;\delta_{0}=\left\{\begin{array}{ll} \frac{1}{2n_{0}} & \text{if}\;n_{0}\;\text{is}\;\mathrm{odd},\\ \frac{1}{2\left(n_{0}-1\right)} & \text{otherwise}. \end{array}\right.$$

In the limit case where $v_{L}\in \left[\frac{1}{2}-\delta_{0};\frac{1}{2}\right]$ and $v_{U}\in \left[\frac{1}{2};\frac{1}{2}+\delta_{0}\right]$, it is easy to prove that both average‐ and median‐based classifications are identical. Otherwise, we can compare the sensitivity and specificity of the average‐based and median‐based classifiers.

Theorem 1 *Assume* (H1) *and* (H3). *We have*

$$\text{sensitivity}\left({\hat{T}}_{A,i}\right)\leq \text{sensitivity}\left({\hat{T}}_{M,i}\right).$$

*The above inequality is strict if and only if* $v_{U}>1/2$, *becoming* $v_{U}>1/2+\delta_{0}$. *Moreover, we have*

$$\text{specificity}\left({\hat{T}}_{A,i}\right)\leq \text{specificity}\left({\hat{T}}_{M,i}\right)$$

*The above inequality is strict if and only if* $v_{L}<1/2$, *becoming* $v_{L}<1/2-\delta_{0}$.

*As a consequence, the median‐based classifier is also better in terms of the misclassification rate and informedness*.

The proof is given in Appendix B: Proof of Theorem 1. The theorem states that the median‐based classifier is better than the average‐based classifier regarding sensitivity and specificity when we introduce the inclusive response, that is, as soon as $v_{L}<0.5<v_{U}$. Both classifiers reflect the properties of their respective scoring. Hence, Theorem 1 yields a first conclusion on the efficiency of the scoring methods in favor of the median‐based scoring.

We also obtained efficiency results on the Bayesian classifier. To state it, we must refer to the loss function $\ell \left(t,\hat{t}\right)$ defined in Section 3.1. Bayesian statistics, which is well grounded in decision theory, is known to be efficient in the sense that it provides the best possible estimators and classifiers given the data if the statistics are computed wisely with the posterior distribution [22]. Here, we can prove the following results.

Theorem 2 *Assume* (H1) *and* (H4). *Consider any classifier* ${\hat{T}}_{i}$ *of the* i *th individual that returns a decision in* {0,1/2,1}, *based on the data* $S_{1},\ldots ,S_{n}$.

- *(Optimality of the likelihood‐based classifier) Whatever the values of* $\left(\theta_{T},p,q\right)$, *we have*

$$E\;\left(\ell \left(T_{i},{\hat{T}}_{L,i}\left(\theta_{T},p,q\right)\right)\right)\leq E\;\left(\ell \left(T_{i},{\hat{T}}_{i}\right)\right).$$

ii *(Bayesian optimality of the Bayesian classifier) We have*

$$E_{\pi }\left(\ell \left(T_{i},{\hat{T}}_{B,i}\right)\right)\leq E_{\pi }\left(\ell \left(T_{i},{\hat{T}}_{i}\right)\right).$$

iii *(Admissibility of* ${\hat{T}}_{B,i}$ *) If on a set of values of* $\left(\theta_{T},p,q\right)$ *with positive prior probability we have*

$$E\;\left[\ell \left(T_{i},{\hat{T}}_{i}\right)\right]<E\;\left[\ell \left(T_{i},{\hat{T}}_{B,i}\right)\right],$$

*then there exists another set of values of* $\left(\theta_{T},p,q\right)$ *with positive prior probability for which the inequality is reversed (strictly)*.

The proof is given in Appendix B: Proof of Theorem 2. Note that E is the expected value given the fixed parameters $\theta_{T}$, p, and q, whereas $E_{\pi }$ integrates them according to the prior distribution of density π. Item (i) states that the likelihood‐based classifier is optimal regarding risk, that is, expected loss at fixed values of $\theta_{T}$, p, and q. Item (ii) considers the Bayesian risk, which is the expected loss integrated over the prior distribution of the fixed parameters. The Bayesian classifier is optimal in this sense. As stated in item (iii), the Bayesian classifier is admissible: no other classifier can outperform it uniformly over the entire set of fixed parameter values. Because of these results, we recommend using the Bayesian classifier in practice.

### 3.3 Accuracy of the Prevalence Estimators

As defined in Equation (8), we have four prevalence estimators: the average‐based, the median‐based, the MAP, and the Bayesian prevalence estimators. The latter ${\hat{\theta }}_{T,B}$ is the expected value of the posterior distribution of the prevalence $\theta_{T}$ given the data. In contrast, the former three ${\hat{\theta }}_{T,K}$, with K={A,M,MAP}, are the empirical means of the associated scores $Y_{K,i}$ for i=1,⋯N.

We first consider the two empirical means ${\hat{\theta }}_{T,A}$ and ${\hat{\theta }}_{T,M}$. They are heavily biased, with a bias that does not tend to 0 as the number of individuals increases. On the other hand, their variances are proportional to 1/n, see Appendix D: Variance of the Prevalence Estimates. Thus, asymptotically, their squared biases dominate their mean squared errors. Moreover, we can compare their bias as follows:

Theorem 3 *(Bias of* ${\hat{\theta }}_{T,A}$ *and* ${\hat{\theta }}_{T,M}$ *) Assume* (H1) *and set* $n_{0}=\min\left\{n_{i},i=1,\ldots ,N\right\}$.

*For any values of* p *and* q *in* (0,1/2), *there exists an interval* J *that contains* p/(p+q) *such that*

$$|E\;\left({\hat{\theta }}_{T,M}\right)-\theta_{T}|\leq |E\;\left({\hat{\theta }}_{T,A}\right)-\theta_{T}|,$$

*except if* $\theta_{T}\in J$.

*The bias of* ${\hat{\theta }}_{T,A}$ *is not influenced by the numbers of replicates* $n_{i}$'*s. Whereas, as* $n_{0}\to \infty$, *then the bias of* ${\hat{\theta }}_{T,M}$ *tends to* 0 *and the length of the interval* J *tends to* 0.

The proof is given in Appendix D: Proof of Theorem 3. The theorem states that the median‐based prevalence estimator ${\hat{\theta }}_{T,M}$ is better than the average‐based prevalence estimator ${\hat{\theta }}_{T,A}$ in terms of bias, except when $\theta_{T}$ is in an interval J. And the length of J is small when the number of replicates is always large. In the latter case, we can rely on the median‐based ${\hat{\theta }}_{T,M}$ to estimate the prevalence. Otherwise, both estimators are heavily biased and should be used with caution.

As always, Bayesian statistics come with its efficiency. In terms of mean squared error, we can prove the following results on the Bayesian prevalence estimator ${\hat{\theta }}_{T,B}$.

Theorem 4 *Assume* (H1), *and consider an estimator* ${\hat{\theta }}_{T}$ *of* $\theta_{T}$, *that is to say any function of the data* $S_{1},\ldots ,S_{N}$.

- *(Bayesian optimality of* ${\hat{\theta }}_{T,B}$ *) We have*

$$E_{\pi }\left[{\left({\hat{\theta }}_{T,B}-\theta_{T}\right)}^{2}\right]\leq E_{\pi }\left[{\left({\hat{\theta }}_{T}-\theta_{T}\right)}^{2}\right].$$

ii *(Admissibility of* ${\hat{\theta }}_{T,B}$ *) If on a set of values of* $\left(\theta_{T},p,q\right)$ *with positive prior probability we have*

$$E\;\left[{\left({\hat{\theta }}_{T}-\theta_{T}\right)}^{2}\right]<E\;\left[{\left({\hat{\theta }}_{T,B}-\theta_{T}\right)}^{2}\right],$$

*then there exists another set of values of*

$\left(\theta_{T},p,q\right)$ *with positive prior probability for which the inequality is reversed (strictly)*.

The proof is given in Appendix D: Proof of Theorem 4. The above Theorem states that ${\hat{\theta }}_{T,B}$ is optimal regarding Bayesian $L^{2}$‐risk, which is the mean squared error integrated over the prior distribution. Moreover, the Bayesian estimator is admissible, which means that there is no other statistic whose mean squared error is always smaller than the one of ${\hat{\theta }}_{T,B}$ whatever the values of the fixed parameters. Additionally, the Bayesian methodology evaluates the uncertainty of the estimated value with credible intervals. These arguments favor the Bayesian prevalence estimator and recommend its use in practice.

## 4 Numerical Results

In this section, we evaluate and compare the four proposed methods based on two primary criteria: the bias in prevalence estimation and the classification quality. The assessment utilizes both simulated datasets—generated under the statistical model and beyond it—and real‐world datasets derived from medical applications. To evaluate the performance of a method on a specific dataset, we use the empirical ${\bar{\ell }}_{a}$‐risk, defined as follows:

$${\bar{\ell }}_{a}‐\text{risk}=\frac{1}{N}\sum_{i=1}^{N}\ell_{a}\left(t_{i},{\hat{t}}_{i}\right),$$

that is, the average of the losses we commit with all decisions taken for each observation i, where the loss function $\ell_{a}$ was defined in Section 3.1.

### 4.1 Some Simulations

#### 4.1.1 Simulations From the Specified Statistical Model

Simulations are based on the statistical model given in Sections 2.1 and 2.2, where p=0.1,q=0.05 and $n_{i}$'s are sampled through a following uniform distribution $n_{i}∼U_{\left[\;\left[2,6\right]\;\right]}$ and the Table 1 details how priors are built.

**TABLE 1:** Hyperparameters values of two different priors used to analyze the simulations.

	$a_{\mathrm{FP}}$	$b_{\mathrm{FP}}$	$a_{\mathrm{FN}}$	$b_{\mathrm{FN}}$	$a_{T}$	$b_{T}$
Default prior	2	2	2	2	0.5	0.5
Misguided prior	50	50	50	50	0.5	0.5

*Note:* The first one is the default prior given in 2.5. The second one is an informative prior that is badly chosen on purpose. See Figure 1 for a description of the prior.

**FIGURE 2:**
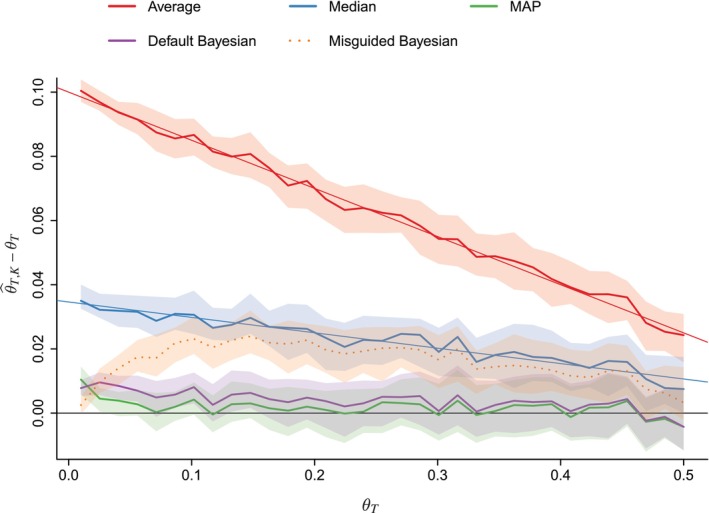
Bias in prevalence estimation for simulated datasets versus $\theta_{T}\in \left[0.01,0.5\right]$. Medians are plotted in thick lines, and [0.4,0.6]− quantile areas are filled with shaded colors, four datasets have been sampled for each value given to $\theta_{T}$. For average and median‐based approaches, the theoretical bias, computed in Appendix D: Proof of Theorem 3, are plotted as thin straight lines. The horizontal black line corresponds with the objective: null error on estimating $\theta_{T}$.

First, we compare the quality of estimating $\theta_{T}$ through the different approaches. Parameter $\theta_{T}$ is evenly sampled between 0.01 and 0.5. For each $\theta_{T}$, datasets of size N=200, have been sampled. Figure 2 gives the results of estimating the parameter $\theta_{T}$, where ${\hat{\theta }}_{T,K}$ is one of the estimators produced by any of the considered approaches. Medians (thick lines) and [0.4,0.6]‐quantiles (shaded areas) are represented. The closer each curve is to the straight black line, the better the approach is. As expected from Theorem 3, the sample mean of median scores produces better prevalence estimates than the sample mean of average scores. Moreover, the bias obtained on simulations with the average‐based and the median‐based approaches follows the linear bias expected from Appendix D: Proof of Theorem 3, plotted as thin straight lines. Furthermore, MAP outperforms other approaches. The default Bayesian is close to these optimal methods, followed by the median‐based approach. On the contrary, the prevalence value inferred by the average‐based method is the worst‐performing.

**FIGURE 3:**
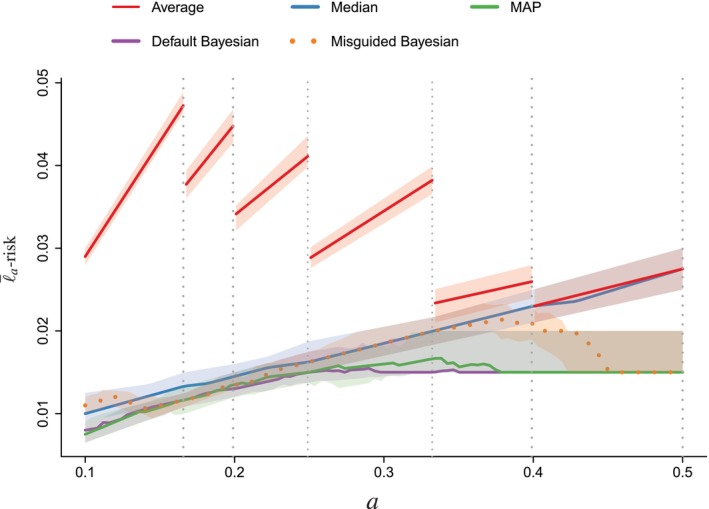
Comparison of all methods when the decision cost a varies and $\theta_{T}=0.4$. Their performance is measured in empirical ${\bar{\ell }}_{a}$‐risk. We run the methods on 300 simulated datasets for a∈[0.1,0.5]. The plain line is the median of the 300 empirical risks, and the band represents the [0.4,0.6]‐quantile interval. The average‐risk jumps when a is equal to an observed value $\frac{s_{i}}{n_{i}}$, where $n_{i}\in \left[\;\left[2,6\right]\;\right]$. In other words, jumps occur when a crosses $\left\{\frac{1}{6};\frac{1}{5};\frac{1}{4};\frac{1}{3};\frac{2}{5}\right\}$, represented as vertical dotted lines.

Figure 3 shows the ${\bar{\ell }}_{a}$‐risks, for $\theta_{T}=0.4$, through their median and their [0.4,0.6]‐quantile area in shaded. As expected from Appendix B: Average and Median‐Based Empirical Risks, the sample mean of median‐risks is linear and increasing, whereas the sample mean of average‐risks is linear and increasing, piecewise, where jumps occur at all the observed values of $\frac{s_{i}}{n_{i}}<0.5$, when $n_{i}$ takes values in [[2,6]]. In our simulations, the average‐based approach gives the poorest results. The Bayesian and MAP approaches are always better than the average and the median solutions. The default Bayesian performs better than the misguided.

In Section 3.3, we recommended using the Bayesian prevalence estimator. The numerical results are in agreement with this recommendation.

#### 4.1.2 Limit Case: Single Measurements

In practice, some individuals may be associated with a single measurement, providing minimal information about their latent state. We conducted additional simulations to assess how the presence of poorly replicated individuals affects performance in more realistic scenarios. We generated datasets of size *N* = 200, varying the proportion of individuals with a single measurement from 100% (all with one measurement) to 0% (all with multiple measurements). The remaining individuals had their number of replicates $n_{i}$ sampled from the discrete Uniform distribution $U_{\left[\;\left[2,6\right]\;\right]}$. For each configuration, we evaluated both classification performance using the empirical $\ell_{a}$‐risk (with *a* = 0.45) and the bias in estimating the prevalence $\theta_{T}$. Figures and detailed results are given in Appendix E: Limit Case: Single Measurements.

**FIGURE E4:**
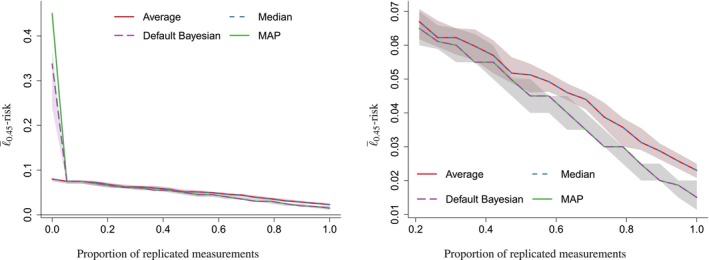
Comparison of empirical risk ${\bar{\ell }}_{a}$‐risk, with a=0.45, when the proportion of replicated measurements varies from 0 (all with one measurement) to 1 (all with multiple measurements). We run the methods on 100 simulated datasets of size N=200, where $\theta_{T}=0.4,p=0.1,q=0.05$ and $n_{i}$ is either 1 (single replicate) or sampled in $U_{\left[\;\left[2,6\right]\;\right]}$ (multiple replicates). Medians are plotted in thick lines, and [0.4,0.6]‐quantile areas are filled with shaded colors. In the right panel, we zoom on configurations where the proportion of individuals with replicated measurements exceeds 20%.

**FIGURE E5:**
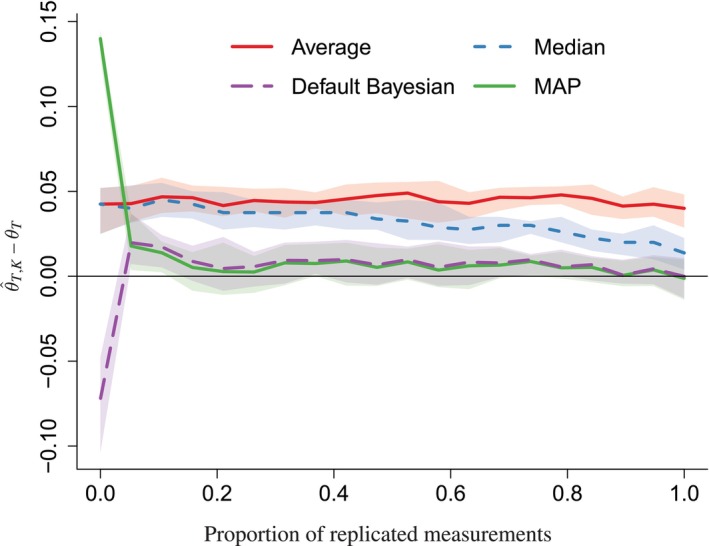
Comparison of the bias ${\hat{\theta }}_{T,K}-\theta_{T}$ in estimating prevalence $\theta_{T}$, when the proportion of replicated measurements varies from 0 (all with one measurement) to 1 (all with multiple measurements). We run the methods on 100 simulated datasets of size N=200, where $\theta_{T}=0.4,p=0.1,q=0.05$ and $n_{i}$ is either 1 (single replicate) or sampled in $U_{\left[\;\left[2,6\right]\;\right]}$ (multiple replicates). This corresponds to *Case A* in Section 4.1.2. Medians are plotted in thick lines, and [0.4,0.6]‐quantile areas are filled with shaded colors.

The results show that all methods perform poorly when all individuals have only one measurement. As soon as a moderate proportion of individuals with multiple replicates is introduced (at least 10%), in Figures E4 and E5 we see that the Bayesian and MAP‐based methods recover much of their performance. In contrast, the average‐based and median‐based methods remain structurally biased and display consistently higher classification errors. Following the theoretical results in Section 3 that assume that at least some $n_{i}$ are larger than three, these findings underscore the importance of including sufficiently replicated individuals in the data to enable meaningful inference.

#### 4.1.3 Limit Case: Heterogeneous Error Rates

To assess the robustness of the proposed methods under heterogeneous measurement error, we considered a setting where the false positive and false negative rates vary across individuals. Specifically, for each individual i, the error rates $p_{i}$ and $q_{i}$ were generated independently according to a logistic transformation of Gaussian noise:

$$\begin{array}{ll} & \text{draw}\;v_{i}^{\left(p\right)}∼N\left(0,\sigma^{2}\right),\;p_{i}=\frac{0.5}{1+\exp\left(-v_{i}^{\left(p\right)}\right)},\\ & \text{draw}\;v_{i}^{\left(q\right)}∼N\left(0,\sigma^{2}\right),\;q_{i}=\frac{0.5}{1+\exp\left(-v_{i}^{\left(q\right)}\right)}. \end{array}$$

Therefore, both $p_{i}$ and $q_{i}$ remain in the interval (0,0.5), with the degree of variability controlled by the parameter σ, ranging from 0.01 (yielding nearly constant error rates) to 1, which reflects a population with substantial differences in measurement reliability across individuals.

**FIGURE E6:**
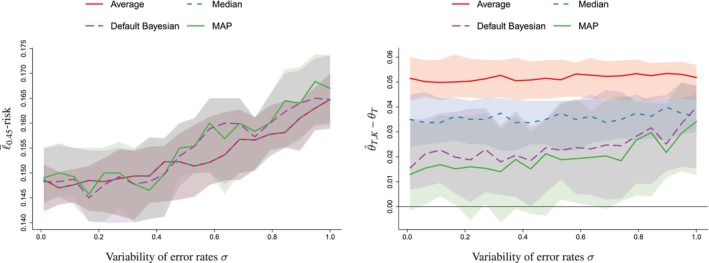
Comparison of methods performance when the variability of error rates σ varies from 0 (homogeneous case) to 1 (strongly heterogeneous case). We run the methods on 100 simulated datasets of size N=200, where $\theta_{T}=0.4$ and $n_{i}$ is sampled in $U_{\left[\;\left[2,6\right]\;\right]}$. Medians are plotted in thick lines, and [0.4,0.6]‐quantile areas are filled with shaded colors. In the left panel, we display the empirical risk ${\bar{\ell }}_{a}$‐risk, with a=0.45 whereas the bias in estimating the prevalence is given in the right panel.

Figure E6 reports the performance of the methods as σ increases, using both the empirical $\ell_{0.45}$‐risk (left) and the bias in prevalence estimation (right). All methods demonstrate relatively stable classification performance with moderate heterogeneity; however, as the variability increases, there is a gradual rise in risk. As for prevalence estimation, the MAP and Bayesian methods outperform the average and median‐based approaches, consistently showing lower bias across all levels of variability.

These results confirm that the proposed methods maintain strong performance even in settings that violate the model assumptions. This robustness is crucial for practical applications, where homogeneity of measurement error across individuals is rarely guaranteed.

### 4.2 A Periodontal Dataset

**FIGURE 4:**
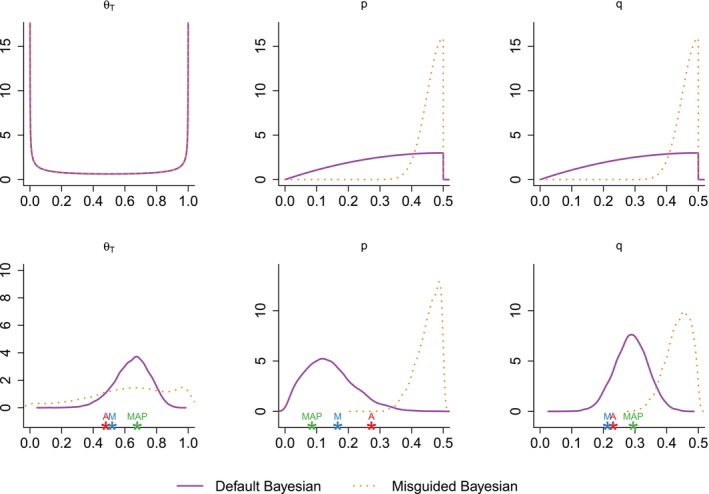
Comparison of the prior distributions (first row) and the associated posterior distributions (second row). From the latent status of each patient, we infer the parameters ${\hat{\theta }}_{T,\text{latent}}=0.58,\;{\hat{p}}_{\text{latent}}=0.187$ and ${\hat{q}}_{\text{latent}}=0.338$. The parameters ${\hat{\theta }}_{T,K},\;{\hat{p}}_{K}$ and ${\hat{q}}_{K}$, estimated by non‐Bayesian methods (*K* = A, M, MAP) are displayed below the respective graphs with stars.

We tried the proposed methods on the periodontal dataset [6] which contains N=50 individuals. The number of replicates per individual, $n_{i}$, varies from 1 to 6. The status variable of the dataset provides a trustworthy diagnostic (*healthy* or *infected*). We used it as the actual value of $T_{i}$. We ran (1) the average‐based method, (2) the median‐based method, (3) the MAP estimator approximation as in Algorithm 1, (4) the default Bayesian method with the default prior given in Section 2.5, which is weakly informative and (5) a misguided Bayesian method, with a poorly chosen informative prior. The prior and posterior distributions of Bayesian methods on the fixed parameters are displayed in Figure 4.

Since we are in a unique situation where the latent status of each patient, denoted as $T_{i}$, is known, it becomes possible to infer the theoretical parameters $\theta_{T}$, p, and q as follows:

(14) $${\hat{\theta }}_{T,\text{latent}}=\frac{1}{N}\sum_{i=1}^{N}T_{i},\;{\hat{p}}_{\text{latent}}=\frac{\sum_{i=1}^{N}S_{i}\left(1-T_{i}\right)}{\sum_{i=1}^{N}n_{i}\left(1-T_{i}\right)}\;\text{and}\;{\hat{q}}_{\text{latent}}=\frac{\sum_{i=1}^{N}\left(n_{i}-S_{i}\right)T_{i}}{\sum_{i=1}^{N}n_{i}T_{i}}.$$

We can also infer the prevalence in the data set as ${\hat{\theta }}_{T,\text{latent}}=29/50=0.58$, the false positivity rate as ${\hat{p}}_{\text{latent}}=9/48=0.187$ and the false negativity rate as ${\hat{q}}_{\text{latent}}=48/142=0.338$. Globally, we see that the default and the misguided Bayesian methods provide very different distributions, illustrating the influence of priors in Bayesian methods. The default Bayesian is almost centered on the values estimated from the latent status $T_{i}$. Indeed, the modes of the default Bayesian posterior density are 0.67,0.12 and 0.29 for $\theta_{T},p$ and q respectively. On the contrary, the misguided Bayesian method provides a peaked posterior distribution for p and q that is wholly shifted towards 0.5 (respective modes are 0.50 and 0.46). In contrast, it provides a uniform posterior distribution for $\theta_{T}$ estimation.

Furthermore, the estimation of parameters p,q and $\theta_{T}$ provided by the average‐based, the median‐based and the MAP methods, computed from Equations (8) and (9), are represented in Figure 4 as points below the graphics. Contrary to Bayesian methods that provide a posterior distribution, these methods offer only a point estimate.

Next, we set the thresholds as $v_{L}=0.45$ and $v_{U}=1-v_{L}$ to compute the classifiers. This means an indecision response is given when the scores are too close to 1/2. We compared classifications to the diagnostics given by the status variable in the confusion matrix of Table 2. Since the thresholds $v_{L}$ and $v_{U}$ are close to 0.5, the average‐based and the median‐based classifiers give the same classification. Indeed, in this example we have $n_{0}=6$, then $a=0.45>1/2-\delta_{0}=0.4$, as defined in Section 3.2.

**TABLE 2:** Classification of the periodontal dataset.

Status	Method	$\hat{T}=0$	$\hat{T}=1/2$	$\hat{T}=1$
Healthy	Average	16	1	4
Healthy	Median	16	1	4
Healthy	MAP	13	0	8
Healthy	Default	13	3	5
Healthy	Misguided	1	7	13
Infected	Average	5	5	19
Infected	Median	5	5	19
Infected	MAP	3	2	24
Infected	Default	3	2	24
Infected	Misguided	1	4	24

	Emp.
Method	$\ell_{0.45}$‐risk
Average	11.7
Median	11.7
MAP	11.9
Default	10.2
Misguided	18.9

*Note:* The table on the left counts how many times a decision has occurred given the method and the status of the individual. The thresholds were set to $v_{L}=a=0.45$ and $v_{U}=0.55$. The table on the right gives the empirical $\ell_{0.45}$‐risk of each method, corresponding to a=0.45.

According to Lemma 1, classification‐accuracy is fitted with the loss $\ell_{0.45}\left(t,\hat{t}\right)$. From the empirical risks given in Table 2, we conclude that the default Bayesian method is the best, and the misguided Bayesian method is the worst. The misguided method has a bad $\ell_{0.45}$‐risk because it diagnoses all patients as infected, whether they are infected or not. Default Bayesian and MAP methods provide the same decisions, except for 3 healthy individuals. For those three individuals, the default method is in favor of 1/2 (inconclusive), whereas the MAP method is in favor of 1. Since predicting 1 for a healthy patient is a larger error than predicting 1/2, the empirical risk of the default Bayesian method (10.2) is smaller than that of the MAP method (11.9).

### 4.3 A Mammogram Screening Dataset

Let us consider N=148 women who may or may not suffer from breast cancer [19, 20]. A gold‐standard diagnostic test gives the breast cancer diagnosis of the patient. Sixty‐four patients are confirmed cancer cases, and 84 are not affected. $n_{\mathrm{rad}}=110$ radiologists read the n mammography films. Further information on this dataset is given in Appendix F: Details of the Mammogram Dataset. Since mammography data are sensitive, they are often proprietary and confidential, generally maintained by the American College of Radiology. An agreement with the ACR is necessary to use complete datasets. We had to run simulations to synthesize complete data with only partial data available.

Moreover, error rates are nonhomogeneous in this dataset, which is out of our model. We have simulated datasets corresponding to this non‐homogeneity of the error rates. The complete description of the simulation procedure is given in Appendix F: A Simulation Procedure for Synthetic Mammography Datasets. In mammograms, authorizing a diagnosis of uncertainty is crucial, as it may correspond to situations where radiologists disagree or mammogram images are challenging to interpret. These cases correspond to situations where clinical follow‐up (additional tests such as biopsies or a second reading by specialists) is preferable to a categorical decision.

**FIGURE 5:**
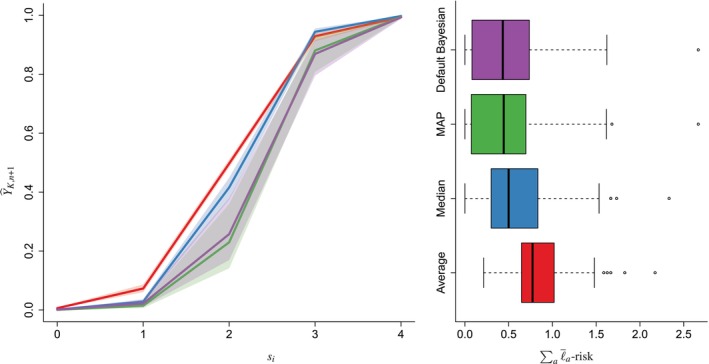
Comparison of prediction scores ${\hat{Y}}_{K,n+1}$. In the left subfigure, medians (lines) and [0.4,0.6]‐quantiles (shaded areas) are represented. In the right subfigure, predictions performance is measured in terms of empirical risk $\sum_{a}{\bar{\ell }}_{a}$‐risk, for a varying from 0.15 to 0.45. We run the methods on 100 simulated mammography datasets subsampled with 4 remaining radiologists.

We simulated 100 datasets with a number of radiologists set to $n_{i}=4$. For each simulation, we used 90% of the 110 mammograms to estimate $\left(\theta_{T,K},p_{K},q_{K}\right)$ for K=A,M,MAP and B. Next we computed the predictions ${\hat{Y}}_{K,n+1}$, as described in Equations (11) and (12), for the 15 remaining mammograms. Figure 5 displays the prediction scores versus the observed number of positive replicates $s_{i}=0,1\cdots ,4$. For each simulation, we additionally compute $\sum_{a}{\bar{\ell }}_{a}$‐risk, for a varying from 0.15 to 0.45. The default Bayesian and the MAP methods outperform the others, whereas the average‐based method provides the largest empirical risk.

## 5 Conclusion and Perspectives

We proposed alternative methods (median, MAP, and Bayesian scoring) to the classical method of averaging binary technical replicates. We allowed for an “indecisive” outcome when the evidence is insufficient, a crucial aspect in medical diagnostics. These alternatives lead to more nuanced classifications. The theoretical analysis and empirical results from both simulated and real‐world medical datasets enable the comparison of the proposed methods. The average‐based approach is intuitive and easy to compute, but it suffers from structural bias in prevalence estimation and poor classification performance. The median‐based method provides a simple yet more robust alternative to the average, particularly when handling indecision responses, and generally enhances classification accuracy. However, it remains biased when the number of replicates is small, as its bias does not vanish asymptotically unless the number of replicates increases. We also proposed a maximum penalized‐likelihood approach, which functions as a MAP method. It improves performance by optimizing a penalized likelihood, resulting in improved classification and estimation accuracy. Nevertheless, it relies on iterative optimization via the EM algorithm, which is sensitive to initialization and may converge to local optima. Finally, the Bayesian approach provides a comprehensive framework for incorporating prior knowledge and quantifying uncertainty. The Bayesian method consistently outperforms the other methods by integrating uncertainty over parameters and returning posterior distributions and credible intervals. Credible intervals not only offer point estimates but also a measure of confidence, which is essential for informed decision‐making in clinical practice and epidemiological studies.

Beyond the idealized modeling framework, we explored two challenging scenarios that are common in real‐world applications. First, when most individuals are associated with a single measurement, our results confirm that no method performs well unless a sufficient fraction of individuals (at least 10%–20%) are associated with three or more replicates. In such cases, only the Bayesian and MAP methods recover meaningful performance. Second, we evaluated robustness to heterogeneous measurement error across individuals. While classification error increases only gradually with variability, the Bayesian and MAP estimators outperform the others in terms of bias, preserving reliable prevalence estimates even under substantial heterogeneity. Taken together, these findings reinforce our recommendation to prefer Bayesian or MAP approaches whenever replication is limited or measurement reliability is suspected to vary. These methods constitute principled approaches to account for uncertainty and model deviations, which are often unavoidable in medical and environmental contexts.

Moreover, the predictive component of our methods provides easy‐to‐use guiding rules for practitioners to get a confident diagnostic based on newly observed data and the information drawn from the dataset. Indeed, a simple table indicates the predictive classification based on the number of replicates and the number of positive replicates in the new data. Once again, the Bayesian method stands out for its better predictive ability, which is crucial in sensitive medical contexts where the cost of an erroneous diagnosis is high. Additionally, the user‐friendly R package implementing these methods will facilitate their wider adoption and contribute to more robust and reliable analyses of binary replicate data.

Our methods are applicable in many biomedical and environmental contexts, where binary decisions derived from repeated tests are subject to significant technical variability, often in the absence of a reliable gold standard. Our methodology, which reconciles binary replicates while accounting for uncertainty and allowing for indecision responses, is well suited to such situations. A first example concerns the assessment of bacterial resistance to antibiotics. For a given bacterial strain, multiple susceptibility tests (e.g., antibiograms) are typically performed to determine whether the strain is resistant (1) or susceptible (0) to a particular antibiotic. These tests may be repeated within or across laboratories, and results may be inconsistent, particularly when the minimum inhibitory concentration is near the clinical breakpoint. In this context, the absence of a definitive reference method and the potential asymmetry of error rates (e.g., rare false positives but frequent false negatives) make our approach particularly relevant. A second application arises in environmental microbiology, particularly in the detection of pathogenic organisms such as *E. coli* or Salmonella in water or food samples. Each sample is typically tested using several binary PCR assays, and test results may vary due to differences in protocols or low concentrations of the target organism. These binary technical replicates require statistical inference methods that can robustly integrate uncertainty to determine whether a sample is contaminated. Finally, high‐throughput toxicity screening in cellular or pharmacological assays provides an ideal setting for our method. Each candidate compound is tested in multiple wells or replicates, with a binary outcome (toxicity observed or not). Our tools enhance the classification of compounds by aggregating technical replicates, while also providing a reliable estimate of toxicity prevalence—an asset in early‐phase drug discovery and compound prioritization.

This study represents a first step towards theoretical results and has not yet incorporated any covariates. Future research will consider covariates to classify individuals better, even if experts use some of them to inform their decisions, rather than focusing solely on the decisions derived from these covariates. This approach would help reduce the variation among different experts or laboratories, a common issue observed, for instance, in multi‐expert annotation of medical images, assessment of bacterial resistance to antibiotics, pathogen detection within environmental microbiology, and laboratory tests for cellular or pharmacological toxicity… In conclusion, both theoretical analysis and empirical results confirm the superior performance of median‐based, MAP and Bayesian alternatives to the average in terms of accuracy and reliability. The Bayesian method offers more cautious and reliable classifications, especially valuable in medical decision‐making contexts, and robust prevalence estimation, making it our recommended choice in practice.

## Author Contributions

All the authors contributed equally.

## Funding

The authors have nothing to report.

## Conflicts of Interest

The authors declare no conflicts of interest.

## Figures and Tables

**FIGURE A1:**
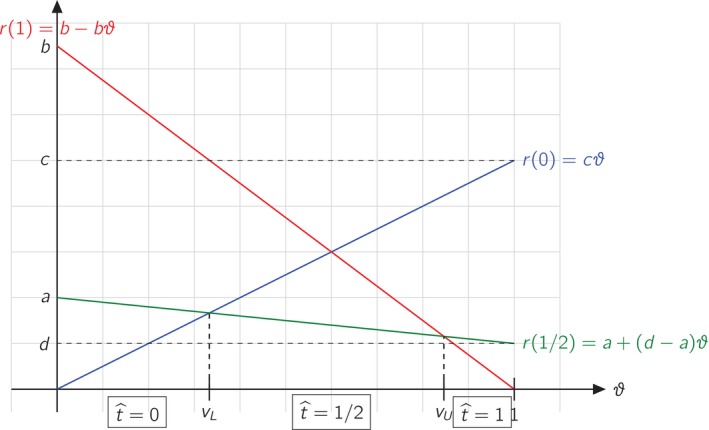
Risks $r\left(\hat{t}\right)$ of deciding $\hat{t}=0,1/2$ or 1 as a function of ϑ. When $\vartheta \in \left(0,v_{U}\right)$, the lowest risk is r(0), thus the best decision is $\hat{t}=0$. When $\vartheta \in \left(v_{L},v_{U}\right)$, the lowest risk is r(1/2), thus the best decision is $\hat{t}=1/2$. When $\vartheta \in \left(v_{U},1\right)$, the lowest risk is r(1), thus the best decision is $\hat{t}=1$.

**FIGURE A2:**
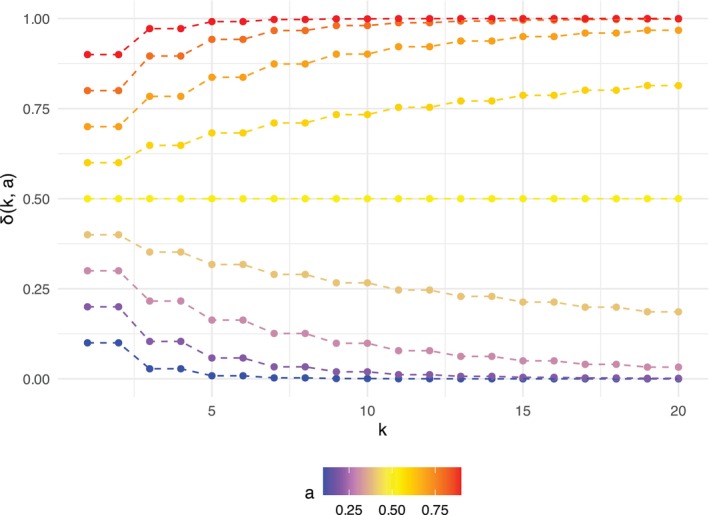
The values of δ(k,a) for a=0.1,0.2,…,0.9, as defined in Equation (A7).

**FIGURE D3:**
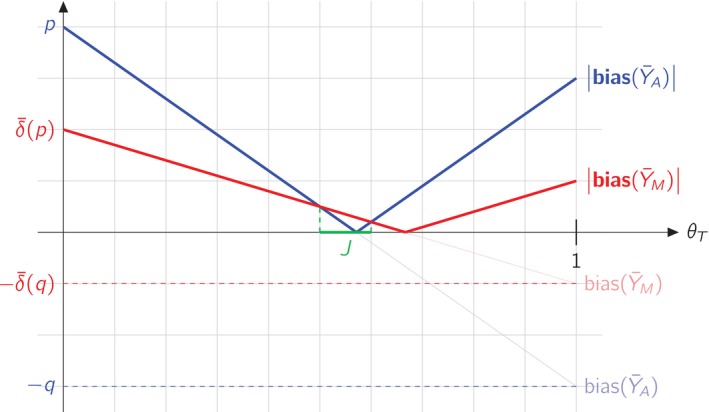
Typical behavior of the Biases of the average‐ and median‐based prevalence estimates ${\hat{\theta }}_{T,A}$ and ${\hat{\theta }}_{T,M}$ as a function of $\theta_{T}$, and their absolute values. In absolute values, the bias of the average‐based estimate in thick blue is smaller than the bias of the median‐based one if and only if $\theta_{T}\in J$.

**FIGURE F7:**
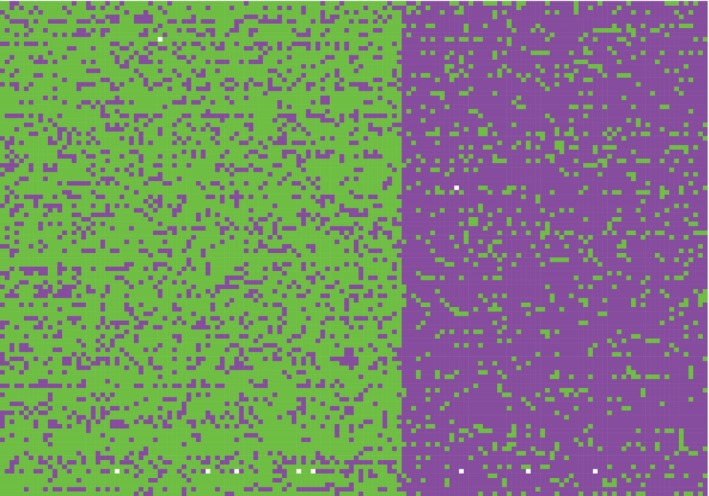
Example of a simulated dataset for the mammography analysis where green pixels correspond to 0's, purple pixels correspond to 1's, and white corresponds to NA's. Each row is a radiologist, and each column is a mammogram.

## Data Availability

The data that support the findings of this study are openly available in Github at https://github.com/pierrepudlo/BinaryReplicates.

## Appendix A A Technical Results

### Full Description of the MAP EM Estimation Algorithm

Recall the likelihood according to Equation (5):

$$ℒ\left(\theta_{T},p,q|s_{1},\ldots ,s_{n}\right)=\prod_{i=1}^{n}\left(\theta_{T}{\left(1-q\right)}^{s_{i}}q^{n_{i}-s_{i}}+\left(1-\theta_{T}\right)p^{s_{i}}{\left(1-p\right)}^{n_{i}-s_{i}}\right).$$

This function's maximization might stick to border points, corresponding to $\hat{p}=0$ or $\hat{q}=0$, for example. Those points are not expected, and in order to avoid them, we have chosen to use priors on both p and q such that

$$\begin{array}{l} p∼B\;\mathrm{eta}{\left(a_{\mathrm{FP}},b_{\mathrm{FP}}\right)}_{|p\in \left(0,1/2\right)}\\ q∼B\;\mathrm{eta}{\left(a_{\mathrm{FN}},b_{\mathrm{FN}}\right)}_{|p\in \left(0,1/2\right)}, \end{array}$$

corresponding to weakly informative priors. The posterior distribution of the parameters is thus proportional to

(A1)
$$\begin{array}{ll} & \pi \left(\theta_{T},p,q,s_{1},\ldots ,s_{n}\right)\propto p^{a_{\mathrm{FP}}-1}{\left(1-p\right)}^{b_{\mathrm{FP}}-1}q^{a_{\mathrm{FN}}-1}{\left(1-q\right)}^{b_{\mathrm{FN}}-1}\\ & \;\prod_{i=1}^{n}\left(\theta_{T}{\left(1-q\right)}^{s_{i}}q^{n_{i}-s_{i}}+\left(1-\theta_{T}\right)p^{s_{i}}{\left(1-p\right)}^{n_{i}-s_{i}}\right). \end{array}$$

The following details the EM algorithm to maximize the problem's likelihood. Rather than maximizing the likelihood, the objective is to maximize the posterior distribution of the parameter, but the EM algorithm can be used the same way.

The EM algorithm is an iterative procedure alternating between the Estimation (E) and Maximization (M) steps. The E‐step computes the expected value of the latent variables $T_{i}$'s given the observed data $s_{i}$'s and the current values of the fixed parameters. The M‐step maximizes the completed posterior distribution of the fixed parameters given the expected values of the latent variables. The completed log‐posterior distribution of the fixed parameters $\theta_{T}$, p, and q given the observed data $\left(s_{1},\ldots ,s_{n}\right)$ and the latent $\left(y_{1},\ldots ,y_{n}\right)$ can be extracted from (A5) and is, up to an additive constant,

We can easily optimize the above completed log‐posterior distribution in $\theta_{T}$, p and q given the values of $\left(s_{1},\ldots ,s_{N}\right)$ and $\left(y_{1},\ldots ,y_{N}\right)$. The M‐steps of the EM algorithm are explicit and incorporate the prior Beta $\left(a_{\mathrm{FP}},b_{\mathrm{FP}}\right)$ on p and Beta $\left(a_{\mathrm{FN}},b_{\mathrm{FN}}\right)$ on q:

(A2)
$$\begin{array}{ll} \theta_{T,M‐\text{step}} & =\frac{1}{N}\sum_{i=1}^{N}y_{i},\;p_{M‐\text{step}}=\frac{a_{\mathrm{FP}}-1+\sum_{i=1}^{N}s_{i}\left(1-y_{i}\right)}{a_{\mathrm{FP}}+b_{\mathrm{FP}}-2+\sum_{i=1}^{N}n_{i}\left(1-y_{i}\right)},\\ q_{M‐\text{step}} & =\frac{a_{\mathrm{FN}}-1+\sum_{i=1}^{N}\left(n_{i}-s_{i}\right)y_{i}}{a_{\mathrm{FN}}+b_{\mathrm{FN}}-2+\sum_{i=1}^{N}n_{i}y_{i}}. \end{array}$$

By default, we set $a_{\mathrm{FP}}=b_{\mathrm{FP}}=a_{\mathrm{FN}}=b_{\mathrm{FN}}=2$, in order to remove borders of the interval introducing as less information as possible. The value of $y_{i}$ plugged in the M‐step is the expected value of the latent variable $T_{i}$ given the observed data $s_{i}$ and the current values of the fixed parameters. (Note that those values of $y_{i}$ are now real numbers between 0 and 1 exactly as our different scores.) This is the result of the E‐step in the EM algorithm. It is also explicit: since $T_{i}$ is a binary variable, this conditional expected value is equal to the probability of $T_{i}=1$ given the observed data $s_{i}$ and the current values of the fixed parameters. We can recognize the definition of the likelihood‐based score $Y_{L,i}\left(\theta_{T},p,q\right)$ given in (4). Thus, the E‐step of the EM algorithm is

(A3)
$$y_{iE‐\text{step}}=Y_{L,i}\left(\theta_{T},p,q\right),$$

where we use the last values of the fixed parameters $\theta_{T}$, p, and q available during the iterative procedure.

As with many numerical optimization algorithms, the EM algorithm is sensitive to the initial values. We thus have to run the EM algorithm several times. Each time, we initialize the latent $y_{i}$'s by drawing them at random from

(A4)
$$y_{i}∼B\;\mathrm{eta}\left(s_{i}+1/2,n_{i}-s_{i}+1/2\right).$$

We then run the EM algorithm starting with an M‐step until convergence. We compare the results obtained from the several runs by using the posterior distribution given in (A1), and the best model is chosen.

The posterior distribution of our mixture model in (A1) suffers from the label‐switching problem; see Chapter 1 [21]. Indeed, there is a symmetry in the posterior: we can exchange the 0 and 1 labels in the latent space and get the same posterior value with $\theta_{T}^{*}=1-\theta_{T}$, $p^{*}=1-q$ and $q^{*}=1-p$ as the new fixed parameters. However, we have assumed that the replicates are noisy measurements of the true value $T_{i}$. Therefore, we have set the constraint that the false‐positivity rate p and false‐negativity rate q are smaller than 1/2. This constraint gives us a relabelling of the latent $y_{i}$'s that removes entirely the label‐switching problem when applying the EM algorithm to our mixture model: each time the constraint on p is violated, we use the symmetry described above, switch the 0 and 1 and change the current values of the fixed parameters to $1-\theta_{T}$, 1−q and 1−p and the current values of all $y_{i}$'s to $1-y_{i}$. Algorithm 1 synthesizes the complete estimation procedure.

ALGORITHM 1 Maximum‐A‐Posteriori estimator approximation of the fixed parameters. 1
**for** r∈[[1,R]] **do**
  
∀i∈[[1,N]] , initialize $y_{r,i}$ : $y_{r,i}∼B\;\mathrm{eta}\left(s_{i}+1/2,n_{i}-s_{i}+1/2\right)$

**while** Convergence not reached **do**
   
Perform M‐step:

$$\theta_{T,\mathrm{EM},r}\leftarrow \frac{1}{N}\sum_{i=1}^{N}y_{r,i},\;p_{\mathrm{EM},r}\leftarrow \frac{a_{\mathrm{FP}}-1+\sum_{i=1}^{N}s_{i}\left(1-y_{r,i}\right)}{a_{\mathrm{FP}}+b_{\mathrm{FP}}-2+\sum_{i=1}^{N}n_{i}\left(1-y_{r,i}\right)},\;q_{\mathrm{EM},r}\leftarrow \frac{a_{\mathrm{FN}}-1+\sum_{i=1}^{N}\left(n_{i}-s_{i}\right)y_{r,i}}{a_{\mathrm{FN}}+b_{\mathrm{FN}}-2+\sum_{i=1}^{N}n_{i}y_{r,i}}$$

   
**if** q>1/2 **then**

$$\left(\theta_{T,\mathrm{EM},r},p_{\mathrm{EM},r},q_{\mathrm{EM},r}\right)\leftarrow \left(1-\theta_{T,\mathrm{EM},r},1-p_{\mathrm{EM},r},1-q_{\mathrm{EM},r}\right)$$

**end if**
   
∀i∈[[1,n]] , perform E‐step: $y_{r,i}\leftarrow Y_{L,i}\left(\theta_{T,\mathrm{EM},r},p_{\mathrm{EM},r},q_{\mathrm{EM},r}\right)$

**end while**

**end for**

The best model $\left({\hat{\theta }}_{T,\mathrm{MAP}},{\hat{p}}_{\mathrm{MAP}},{\hat{q}}_{\mathrm{MAP}}\right)$ verifies

$$\left({\hat{\theta }}_{T,\mathrm{MAP}},{\hat{p}}_{\mathrm{MAP}},{\hat{q}}_{\mathrm{MAP}}\right)=\underset{r\in \left[\;\left[1,R\right]\;\right]}{\arg\;\max}\;\pi \left(\theta_{T,\mathrm{EM},r},p_{\mathrm{EM},r},q_{\mathrm{EM},r},s_{1},\ldots ,s_{n}\right)$$

### Bayesian Posterior Calculation

Using the observed value $\left(s_{1},\ldots ,s_{N}\right)$ of the sufficient statistic $\left(S_{1},\ldots ,S_{N}\right)$, the joint density of the Bayesian model is thus, see Figure 1,

(A5)
$$\begin{array}{ll} & \pi \left(\theta_{T},p,q,y_{1},\ldots ,t_{N},t_{1},\ldots ,s_{N}\right)\\ & \;=\pi \left(\theta_{T},p,q\right)\prod_{i=1}^{N}\;P_{\pi }\left(T_{i}=t_{i}|\theta_{T}\right)\;P_{\pi }\left(S_{i}=s_{i}|T_{i}=t_{i},p,q\right)\\ & \;\;=\pi \left(\theta_{T},p,q\right)\prod_{i=1}^{N}\theta_{T}^{t_{i}}{\left(1-\theta_{T}\right)}^{1-t_{i}}\left(\begin{array}{c} n_{i}\\ s_{i} \end{array}\right)\left\{t_{i}{\left(1-q\right)}^{s_{i}}q^{n_{i}-s_{i}}+\left(1-t_{i}\right)p^{s_{i}}{\left(1-p\right)}^{n_{i}-s_{i}}\right\}. \end{array}$$

If we integrate over the latent $T_{i}$'s, we have the joint density of the observed data and the fixed parameters as follows:

(A6)
$$\begin{array}{cc} & \pi \left(\theta_{T},p,q,s_{1},\ldots ,s_{N}\right)\\ & \;=\pi \left(\theta_{T},p,q\right)\prod_{i=1}^{N}\left(\begin{array}{c} n_{i}\\ s_{i} \end{array}\right)\left\{\theta_{T}{\left(1-q\right)}^{s_{i}}q^{n_{i}-s_{i}}+\left(1-\theta_{T}\right)p^{s_{i}}{\left(1-p\right)}^{n_{i}-s_{i}}\right\}. \end{array}$$

Despite this explicit expression of the joint density, the posterior distribution of the fixed parameters $\theta_{T}$, p, and q given the data $\left(s_{1},\ldots ,s_{N}\right)$ is difficult to compute explicitly. We use the R‐package rstan [23], which implements a Hamiltonian Monte Carlo algorithm to sample from the posterior distribution of the fixed parameters $\theta_{T}$, p, and q given the data. Latent values of the $T_{i}$'s are drawn at each iteration of the MCMC algorithm from the predictive distribution of the $T_{i}$'s given the data $X_{\mathrm{ij}}$'s and the current values of the fixed parameters $\theta_{T}$, p, and q. The predictive distribution of the $T_{i}$'s given the data $S_{i}$'s and the current values of the parameters $\theta_{T}$, p, and q is a product of Bernoulli distributions given by (3).

### On the Likelihood‐Based Scores

Lemma 2
*Assume* $\theta_{T}\in \left(0,1\right)$, p∈(0,1/2) *and* q∈(0,1/2). *The likelihood‐based score, defined as in* (4), *is an increasing function of* s.
It is enough to prove that the following function is increasing in s:

$$Y_{L}\left(s\right)=\theta_{T}{\left(\theta_{T}+\left(1-\theta_{T}\right){\left(\frac{1-p}{q}\right)}^{n-s}{\left(\frac{p}{1-q}\right)}^{s}\right)}^{-1}.$$

Actually $s\mapsto {\left(\frac{1-p}{q}\right)}^{n-s}{\left(\frac{p}{1-q}\right)}^{s}$ is a decreasing function of s because

$$\begin{array}{l} {\left(\frac{q}{1-p}\right)}^{n-s+1}\;{\left(\frac{1-q}{p}\right)}^{s-1}<{\left(\frac{q}{1-p}\right)}^{n-s}\;{\left(\frac{1-q}{p}\right)}^{s}\Leftrightarrow \frac{q}{1-p}<\frac{1-q}{p}\\ \;\Leftrightarrow 0<1-\left(p+q\right). \end{array}$$

And the last inequality is true because p<1/2 and q<1/2.
Proof of Lemma 1 Since $r\left(\hat{t}\right)=E\;\left[\ell \left(T,\hat{t}\right)\right]$, we have

The three risks are thus affine functions of ϑ, see Figure A1. The best decision is of the desired form if and only if, as functions of ϑ, the three risks r(0), r(1/2) and r(1) intersect each other as in Figure A1. This happens if and only if the two following conditions are satisfied:

- The point where the risks r(0) and r(1) intersect is above the line of r(1/2).
- The slope of r(1/2) is between those of r(0) and r(1), that is to say in (−b,c).

Condition (ii) is clearly equivalent to −b<(d−a)<c. Moreover, the point at which r(0) and r(1) intersect has coordinates

$$\left(\frac{b}{b+c},\frac{\mathrm{bc}}{b+c}\right).$$

And at ϑ=b/(b+c), the risk r(1/2) is equal to a+(d−a)b/(b+c). Hence, condition (i) is equivalent to the first inequality in the Proposition. Finally, the values of $v_{L}$ and $v_{U}$ are obtained by solving the equations r(0)=r(1/2) and r(1)=r(1/2), respectively.

### Properties of the Binomial Distribution

We need to introduce some coefficients related to the Binomial distribution to understand the probabilistic properties of the median‐based scoring. For any integer k and any real a∈(0,1), we set

(A7)
$$\begin{array}{ll} u\left(k,a\right) & =P\;\left(B\;\text{in}\left(k,a\right)>k/2\right),\;v\left(k,a\right)=P\;\left(B\;\text{in}\left(k,a\right)=k/2\right)\;\text{and}\\ \delta \left(k,a\right) & =u\left(k,a\right)+v\left(k,a\right)/2. \end{array}$$

Note that, If k is an odd number, then v(k,a)=0. Values of δ(k,a) are given in Figure A2. Proposition 1
*The* δ *‐coefficients have the following properties*.

δ(1,a)=a.

1−δ(k,a)=δ(k,1−a).

- *If* k *is an even number, then* δ(k,a)=δ(k−1,a).
- *If* a<1/2, *then* δ(k,a) *is a non‐increasing function of* k.
- *If* a<1/2, *then* δ(k,a) *tends to* 0 *when* k→∞.

We introduce $B_{i}$, i=1,2,…, an iid sequence of random variables distributed according to Ber(a), and set $Z_{k}=B_{1}+\cdots +B_{k}∼B\;\text{in}\left(k,a\right)$ for all k≥1.
***Proof of* (i)**
We have $\delta \left(1,a\right)=P\;\left(Z_{1}>1/2\right)+P\;\left(Z_{1}=1/2\right)/2=P\;\left(Z_{1}=1\right)=a$.
***Proof of* (ii)**
Consider first the case where k is odd. We have $1-u\left(k,a\right)=1-P\;\left(Z_{k}>k/2\right)=P\;\left(Z_{k}<k/2\right)$ since k/2 is not an integer. And $P\;\left(Z_{k}<k/2\right)=P\;\left(k-Z_{k}>k/2\right)$. Now $k-Z_{k}=\left(1-B_{1}\right)+\cdots +\left(1-B_{k}\right)∼B\;\text{in}\left(k,1-a\right)$. Thus, 1−u(k,a)=u(k,1−a). Moreover, when k is odd, v(k,a)=0 and δ(a,k)=u(a,k). Hence, (ii) is proven when k is odd. Consider now the case where k is even. We have $1-u\left(k,a\right)=1-P\;\left(Z_{k}>k/2\right)=P\;\left(Z_{k}\leq k/2\right)=P\;\left(Z_{k}<k/2\right)+P\;\left(Z_{k}=k/2\right)$. Like above, $P\;\left(Z_{k}<k/2\right)=u\left(k,1-a\right)$. And $v\left(k,a\right)=P\;\left(Z_{k}=k/2\right)=P\;\left(k-Z_{k}=k/2\right)=v\left(k,1-a\right)$. So 1−u(k,a)=[u(k,1−a)+v(k,1−a)]. Thus,

$$\begin{array}{ll} 1-\delta \left(k,a\right) & =1-u\left(k,a\right)-\frac{1}{2}v\left(k,a\right)=\left[u\left(k,1-a\right)+v\left(k,1-a\right)\right]\\ & \;-\frac{1}{2}v\left(k,1-a\right)=\delta \left(k,1-a\right). \end{array}$$

***Proof of* (iii)**
Set k=2m, where m is an integer. We have $\delta \left(2m-1,a\right)=u\left(2m-1,a\right)=\;P\left(Z_{2m-1}>m-1/2\right)=\;P\left(Z_{2m-1}\geq m\right)$. And $Z_{2m-1}=Z_{2m}-B_{2m}$. Since $B_{2m}$ is either 0 or 1, we have

$$\left\{Z_{2m-1}\geq m\right\}=\left\{Z_{2m}\geq m+1\right\}\cup \left\{Z_{2m}=m,B_{2m}=0\right\},$$

and the two events are disjoint. Thus $u\left(2m-1,a\right)=u\left(2m,a\right)+\;P\left(Z_{2m}=m,B_{2m}=0\right)$. To compute the last probability, recall that $Z_{2m}=B_{1}+\cdots +B_{2m}$, and so, $Z_{2m}=m$ means that exactly half of the $B_{i}$'s are equal to 0. Thus, given $Z_{2m}=m$, the probability that $B_{2m}=0$ is m/(2m)=1/2. Hence $P\;\left(Z_{2m}=m,B_{2m}=0\right)=P\;\left(Z_{2m}=m\right)/2=v\left(2m,a\right)/2$ and finally, u(2m−1,a)=u(2m,a)+v(2m,a)/2.
***Proof of* (iv)**
Set a<1/2. It is enough to prove that, for all m, δ(2m−1,a)=δ(2m,a)>δ(2m+1,a). The equality is the result of (iii). Thus, it is enough to prove that δ(2m,a)>δ(2m+1,a) for all m. Fix any m. We have $\delta \left(2m+1,a\right)=P\;\left(Z_{2m+1}\geq m+1\right)$. And $Z_{2m+1}=Z_{2m}+B_{2m+1}$ with independence between these last two variables. Thus,

$$\delta \left(2m+1,a\right)=\left(1-a\right)\;P\;\left(Z_{2m}\geq m+1\right)+aP\;\left(Z_{2m}\geq m\right).$$

Using $P\;\left(Z_{2m}\geq m\right)=P\;\left(Z_{2m}\geq m+1\right)+P\;\left(Z_{2m}=m\right)$ in the above, we get

$$\delta \left(2m+1,a\right)=P\;\left(Z_{2m}\geq m+1\right)+aP\;\left(Z_{2m}=m\right)=u\left(2m,a\right)+a\;v\left(2m,a\right).$$

Since a<1/2, we have av(2m,a)<v(2m,a)/2 and thus δ(2m+1,a)<δ(2m,a).
***Proof of* (v)**
Since a<1/2, Hoefding's inequality gives

$$P\;\left(Z_{k}\geq k/2\right)\leq \exp\left(-2k{\left(1/2-a\right)}^{2}\right).$$

Thus, $\delta \left(k,a\right)=P\;\left(Z_{k}>k/2\right)+P\;\left(Z_{k}=k/2\right)/2\leq P\;\left(Z_{k}\geq k/2\right)$ tends to 0 when k→∞.

## Appendix B Theoretical Results on the Classifiers

Proof of Theorem 1 The sensitivity of a classifier $\hat{T}$ is $P\;\left(\hat{T}=1|T=1\right)$. The average‐based classifier is one if and only if $S_{i}>n_{i}v_{U}$. And the median‐based classifier is one if and only if $S_{i}>n_{i}/2$. Because of (H3), $\left\{S_{i}>n_{i}v_{U}\right\}\subset \left\{S_{i}>n_{i}/2\right\}$, and thus the median‐based classifier is more sensitive than the average‐based classifier. There is equality if and only if both events are equal. This happens if and only if $v_{U}=1/2$. In the case where all $n_{i}$ are equal, this happens if and only if $v_{U}<1/2+\delta_{0}$, where $\delta_{0}$ is defined in Section 3.2. The same kind of reasoning yields to the inequality on the specificities of the classifiers.
Proof of Theorem 2
***Proof of*** (i)
The likelihood‐based scoring is $P\;\left(T_{i}=1\right)$. Because of (H4) and Lemma 1, the thresholding of this scoring with $v_{L}$ and $v_{U}$ gives the best estimator regarding ℓ‐risk. Hence, the desired inequality. □
***Proof of*** (ii)
We can also apply Lemma 1, using the expected value $E_{\pi }$ given the data $S=\left(S_{1},\ldots ,S_{n}\right)$, with $\vartheta =P_{\pi }\left(T_{i}=1|S\right)$ which is the definition of $Y_{B,i}$. Because of (H4), Lemma 1 gives that ${\hat{T}}_{B,i}$ is the best estimator in terms of ℓ‐posterior risk, that is,

$$E_{\pi }\left(\ell (T_{i},{\hat{T}}_{B,i}\right)|S)\leq E_{\pi }\left(\ell \left(T_{i},{\hat{T}}_{i}\right)|S\right).$$

Integrating over the marginal distribution of S in the Bayesian model yields the desired inequality. □
***Proof of*** (iii)
If (iii) is wrong, then the inequality has a prior probability of 1. That is, with prior probability equal to 1,

$$E_{\pi }\left[\ell \left(T_{i},{\hat{T}}_{i}\right)|\theta_{T},p,q\right]<E_{\pi }\left[\ell \left(T_{i},{\hat{T}}_{B,i}\right)|\theta_{T},p,q\right].$$

If we integrate this over the prior distribution, we get a classifier ${\hat{T}}_{i}$ strictly better than the Bayesian classifier ${\hat{T}}_{B,i}$, which is impossible because of (ii). Hence, (iii) is true.

### Average and Median‐Based Empirical Risks

Let us denote by H the set of the healthy individuals and by I the set of the infected ones. Let us consider a decision cost $a\in ]0,\frac{1}{2}]$. From the definition of the loss function given in Section 3.1, we have

$$\ell_{a}^{M}=a\sum_{i=1}^{N}1\;l_{\frac{S_{i}}{n_{i}}=\frac{1}{2}}+\sum_{i=1}^{N}1\;l_{\frac{S_{i}}{n_{i}}>\frac{1}{2}}1\;l_{i\in H}+\sum_{i=1}^{N}1\;l_{\frac{S_{i}}{n_{i}}<\frac{1}{2}}1\;l_{i\in I}.$$

Then $\ell_{a}^{M}$ is a linear and increasing function of the decision cost a. In a similar way,

$$\ell_{a}^{A}=a\sum_{i=1}^{N}1\;l_{\frac{S_{i}}{n_{i}}\in \left[a,1-a\right]}+\sum_{i=1}^{N}1\;l_{\frac{S_{i}}{n_{i}}\in ]1-a,1]}1\;l_{i\in H}+\sum_{i=1}^{N}1\;l_{\frac{S_{i}}{n_{i}}\in [0,a[}1\;l_{i\in I}.$$

It is obvious that as long as a (or 1−a) does not cross an observable value of Si/ni, the terms involved in the sums are constant, and so the average‐risk $\ell_{a}^{A}$ is linear and increasing with a. Then jumps occur only when a crosses an observed value of $\frac{s_{i}}{n_{i}}$. In this case, the average risk increases from 1−a times the number of infected patients with $\frac{s_{i}}{n_{i}}=a$ but decreases from a times the number of healthy patients with $\frac{s_{i}}{n_{i}}=a$. At the same time, the average risk increases from 1−a times the number of healthy patients with $\frac{s_{i}}{n_{i}}=1-a$ but decreases from a times the number of infected patients with $\frac{s_{i}}{n_{i}}=1-a$. Note that infected patients are mainly expected to have $\frac{s_{i}}{n_{i}}>\frac{1}{2}$ whereas healthy patients are mainly expected to have $\frac{s_{i}}{n_{i}}<\frac{1}{2}$.

## Appendix C Theoretical Results on the Scores

### Bias of the Scores

For an individual i, the two frequentist scores $Y_{A,i}$ and $Y_{M,i}$ are biased when we consider recovering the latent $T_{i}$ variable or the prevalence $\theta_{T}$. Their expected and conditional expected values given $T_{i}$ are explicit; see Proposition 2 below. Proposition 2
*(Bias). Fix an individual* i *for which we observe* $n_{i}$ *replicates. The average‐based scoring* $Y_{A,i}$ *is such that*

$$\begin{array}{ll} E\;\left(Y_{A,i}|T_{i}\right) & =T_{i}\left(1-q\right)+\left(1-T_{i}\right)p,\\ \;E\;\left(Y_{A,i}\right) & =\theta_{T}\left(1-q\right)+\left(1-\theta_{T}\right)p. \end{array}$$

*The median‐based scoring* $Y_{M,i}$ *is such that*

$$\begin{array}{ll} E\;\left(Y_{M,i}|T_{i}\right) & =T_{i}\delta \left(n_{i},1-q\right)+\left(1-T_{i}\right)\delta \left(n_{i},p\right),\\ \;E\;\left(Y_{M,i}\right) & =\theta_{T}\delta \left(n_{i},1-q\right)+\left(1-\theta_{T}\right)\delta \left(n_{i},p\right), \end{array}$$

*with the coefficients defined in Equation* (A7).
For all K∈{A,M}, $E\;\left(T_{K,i}|T_{i}\right)$ is a linear function of $T_{i}$. Thus, computing $E\;\left(T_{K,i}\right)$ from $E\;\left(T_{K,i}|T_{i}\right)$ is straightforward: we replace $T_{i}$ in the expression by its expected value $\theta_{T}$. The two scores are explicit functions of $S_{i}$, given by $Y_{A,i}=S_{i}/n_{i}$ and Equation (2) above. Integrating those formulae over the Binomial distribution of $\left[S_{i}|T_{i}\right]$ in (1) yields the desired results.

### Variance of the Scores

The variance measures the random variability of each score around its expected value. We can compute them explicitly for the average‐ and median‐based scores as follows. To state the results, we need to introduce another coefficient as in Equation (A7):

(C8)
$$\gamma \left(k,a\right)=u\left(k,a\right)\left(1-u\left(k,a\right)\right)+\frac{1}{4}v\left(k,a\right)\left(1-v\left(k,a\right)\right)-u\left(k,a\right)v\left(k,a\right).$$

Proposition 3
*(Variance). Fix an individual* i *for which we observe* $n_{i}$ *replicates. The average‐based scoring* $Y_{A,i}$ *is such that*

$$\begin{array}{ll} \mathrm{var}\left(Y_{A,i}|T_{i}\right) & =T_{i}\frac{q\left(1-q\right)}{n_{i}}+\left(1-T_{i}\right)\frac{p\left(1-p\right)}{n_{i}},\\ \;\mathrm{var}\left(Y_{A,i}\right) & =\theta_{T}\left(1-\theta_{T}\right){\left(1-q-p\right)}^{2}+\theta_{T}\frac{q\left(1-q\right)}{n_{i}}+\left(1-\theta_{T}\right)\frac{p\left(1-p\right)}{n_{i}}. \end{array}$$

*The median‐based scoring* $Y_{M,i}$ *is such that*

$$\begin{array}{ll} \mathrm{var}\left(Y_{M,i}|T_{i}\right) & =T_{i}\gamma \left(n_{i},1-q\right)+\left(1-T_{i}\right)\gamma \left(n_{i},p\right),\\ \mathrm{var}\left(Y_{M,i}\right) & =\theta_{T}\left(1-\theta_{T}\right){\left(1-\delta \left(n_{i},q\right)-\delta \left(n_{i},p\right)\right)}^{2}\\ & \;+\theta_{T}\gamma \left(n_{i},1-q\right)+\left(1-\theta_{T}\right)\gamma \left(n_{i},p\right). \end{array}$$

For the average‐based score $Y_{A,i}$, we used $Y_{A,i}=S_{i}/n_{i}$ and the Binomial distribution of $\left[S_{i}|T_{i}\right]$ in (1) to compute the conditional variance given $T_{i}$. The unconditional variance is obtained by using

$$\mathrm{var}\left(Y_{A,i}\right)=E\;\left(\mathrm{var}\left(Y_{A,i}|T_{i}\right)\right)+\mathrm{var}\left(E\;\left(Y_{A,i}|T_{i}\right)\right).$$

Likewise, for the variance of the median‐based scoring $Y_{M,i}$, using Equation (2).

## Appendix D Theoretical Results on the Prevalence Estimates

### Prevalence Estimates Bias

There is a systematic bias in the average‐based and median‐based prevalence estimates, which are empirical means of their respective scores. Indeed, using Proposition 2, we can compute their bias. We need the following coefficients to state the results: for all a∈(0,1), we set

$$\bar{\delta }\left(a\right)=\frac{1}{n}\sum_{i=1}^{n}\delta \left(n_{i},a\right),$$

with the coefficients introduced in Equation (A7). Proposition 4
*The average‐based prevalence estimate* ${\hat{\theta }}_{T,A}$ *is such that*

$$E\;\left({\hat{\theta }}_{T,A}\right)=\theta_{T}\left(1-q\right)+\left(1-\theta_{T}\right)p.$$

*The median‐based prevalence estimate* ${\hat{\theta }}_{T,M}$ *is such that*

$$E\;\left({\hat{\theta }}_{T,M}\right)=\theta_{T}\left(1-\bar{\delta }\left(q\right)\right)+\left(1-\theta_{T}\right)\bar{\delta }\left(p\right),$$

*Moreover, if (* *H2* *) is true, then*

$$\bar{\delta }\left(p\right)<p\;\text{and}\;\bar{\delta }\left(q\right)<q.$$

Proof of Theorem 3 Fix p and q in (0,1/2) and assume (H2). The bias of the prevalence estimates are the linear functions of $\theta_{T}$ because of Proposition 4:

$$\begin{array}{ll} \;\text{bias}\left({\hat{\theta }}_{T,A}\right) & =E\;\left({\hat{\theta }}_{T,A}\right)-\theta_{T}=p-\theta_{T}\left(p+q\right),\\ \text{bias}\left({\hat{\theta }}_{T,M}\right) & =E\;\left({\hat{\theta }}_{T,M}\right)-\theta_{T}=\bar{\delta }\left(p\right)-\theta_{T}\left(\bar{\delta }\left(p\right)+\bar{\delta }\left(q\right)\right). \end{array}$$

The first one is null at $\theta_{T}=p/\left(p+q\right)$, and the second one is null at $\theta_{T}=\bar{\delta }\left(p\right)/\left(\bar{\delta }\left(p\right)+\bar{\delta }\left(q\right)\right)$. The absolute values of these biases are thus as in Figure D3. And, because of (H2), $\bar{\delta }\left(p\right)<p$ and $\bar{\delta }\left(q\right)<q$. Thus,

$$\left|\text{bias}\left({\hat{\theta }}_{T,M}\right)\right|<\left|\text{bias}\left({\hat{\theta }}_{T,A}\right)\right|$$

except when $\theta_{T}$ is in the interval, we denote J, where the blue curve is below the red one in Figure D3. And, since the blue curve is null at p/(p+q), this value is in J. Because of Proposition 1, δ(k,p) is a non‐increasing function of k, and thus $\bar{\delta }\left(p\right)\leq \delta \left(n_{0},p\right)$. And likewise, $\bar{\delta }\left(q\right)\leq \delta \left(n_{0},q\right)$. Moreover, the two bounds $\delta \left(n_{0},p\right)$ and $\delta \left(n_{0},q\right)$ tends to 0 when $n_{0}\to \infty$. That is to say that the red curve in Figure D3 tends to zero when $n_{0}\to \infty$, whereas the blue curve does not change. Hence, the length of J tends to 0 when $n_{0}\to \infty$.

### Variance of the Prevalence Estimates

The variance of the average‐ or median‐based estimate is the variance of the empirical means of the scores. As for the biases, we can compute them explicitly. We need the following coefficients to state the results: for all a∈(0,1), we set

$$\bar{\gamma }\left(a\right)=\frac{1}{N}\sum_{i=1}^{N}\gamma \left(n_{i},a\right)\;\text{and}\;\tilde{n}={\left(\frac{1}{N}\sum_{i=1}^{N}\frac{1}{n_{i}}\right)}^{-1}.$$

Note that $\tilde{n}$ is the harmonic mean of the $n_{i}$'s. Proposition 5
*The average‐based prevalence estimate* ${\hat{\theta }}_{T,A}$ *is such that*

$$\mathrm{var}\left({\hat{\theta }}_{T,A}\right)=\frac{1}{N}\left\{\theta_{T}\left(1-\theta_{T}\right)\left(1-q+p\right)+\theta_{T}\frac{q\left(1-q\right)}{\tilde{n}}+\left(1-\theta_{T}\right)\frac{p\left(1-p\right)}{\tilde{n}}\right\}.$$

*The median‐based prevalence estimate* ${\hat{\theta }}_{T,M}$ *is such that*

$$\mathrm{var}\left({\hat{\theta }}_{T,M}\right)=\frac{1}{N}\left\{\theta_{T}\left(1-\theta_{T}\right)\left(1-\bar{\delta }\left(q\right)+\bar{\delta }\left(p\right)\right)+\theta_{T}\bar{\gamma }\left(1-q\right)+\left(1-\theta_{T}\right)\bar{\gamma }\left(p\right)\right\}.$$

Both prevalence estimates are empirical means of their independent, respective scores. Thus, the proposed formulae are straightforward consequences of Proposition 3, which gives the variances of the scores. □
Proof of Theorem 4 The proof is similar to that of Theorem 2 given in Proof of Theorem 2. We have to replace the loss function ℓ by the squared error loss function $L^{2}\left(\theta_{T},\hat{\theta }\right)={\left(\theta_{T}-\hat{\theta }\right)}^{2}$. And, to replace Lemma 1, we use the fact that the best estimator in terms of the posterior $L^{2}$‐risk is the posterior mean. Likewise, it achieves the minimum Bayesian $L^{2}$‐risk and is admissible.

## Appendix E Additional Simulations

### Limit Case: Single Measurements

We generated datasets of size *N*=200, varying the proportion of individuals with a single measurement from 100% (all with one measurement) to 0% (all with replicated measurements). The remaining individuals had their number of replicates $n_{i}$ sampled in the discrete Uniform distribution $U_{\left[\;\left[2,6\right]\;\right]}$. For each configuration, we evaluated both classification performance using the empirical $\ell_{a}$‐risk, with *a* = 0.45 and the bias in estimating the prevalence $\theta_{T}$. In Figure E4 we report the empirical $\ell_{a}$‐risk (with a=0.45) as a function of the proportion of individuals with replicated measurements. In Figure E5 we evaluate the bias in estimating the true prevalence $\theta_{T}$ as a function of replicate configuration.

When all individuals are poorly replicated, all methods suffer from high classification risk and are strongly biased, especially the default Bayesian and MAP methods (Figures E4—left—and E5, when the proportion of replicated measurements is close to 0). However, classification performance globally improves as soon as individuals with richer replicate profiles are introduced (Figure E4—right—Zoom on the region where the proportion of well‐replicated individuals exceeds 20%). In Figure E5, we see that the average and median‐based estimators remain biased even when replication improves, confirming the structural bias previously discussed in Section 3.3. In contrast, both MAP and Bayesian estimators converge rapidly to an unbiased regime as soon as a minority of individuals (at least 10%) are associated with multiple replicates.

### Limit Case: Heterogeneous Error Rates

Figure E6 investigates the robustness of the proposed methods when the data are generated outside of our model assumptions, specifically under heterogeneous measurement error. In this setting, individual‐specific false‐positive and false‐negative rates $\left(p_{i},q_{i}\right)$ are sampled independently via logistic transformations of Gaussian noise, with variability controlled by the parameter σ∈[0,1]. When σ=0, the classical homogeneous model is recovered; as σ increases, heterogeneity among individuals becomes more pronounced.

The left panel of Figure E6 displays the empirical $\ell_{0.45}$‐risk. We observe that classification performance remains relatively stable for all methods when variability is moderate (σ<0.4). However, as σ increases beyond this point, all the methods deteriorate steadily.

The right panel of Figure E6 displays the bias in estimating the true prevalence $\theta_{T}$ as a function of the variability σ in individual‐specific error rates. As expected, the average‐based and median‐based estimators show the highest bias. Both the Bayesian and MAP estimators yield the lowest bias, even when the variability in measurement error is substantial. This robustness highlights the advantage of modeling the global structure of the data and incorporating uncertainty over error parameters. It confirms that these methods can provide reliable prevalence estimates even when the underlying assumptions of error homogeneity are violated.

## Appendix F Real Case Data Information

### Details of the Mammogram Dataset

Three radiologists did not consider all the exams: radiologist code 1201 to whom 63 positive cases are presented, corresponding to 1 missing positive case exam. Radiologist 7714 to whom 61 positive cases and 79 negative cases are presented, corresponding to 3 missing positive case exams and five missing negative case exams. Radiologist 9007 to whom 83 negative cases are presented, corresponding to one missing negative case exam. Thus, four missing positive case exams and six missing negative case exams among the $n_{\mathrm{rad}}$ radiologists.

For any radiologist j, it is possible to estimate his false‐positivity rate $p_{j}$, and his false‐negativity rate $q_{j}$ as follows:

(F9)
$${\hat{p}}_{j}=\frac{\sum_{i=1}^{N}1\;l_{T_{i}=0}1\;l_{X_{\mathrm{ij}}=1}1\;l_{m_{\mathrm{ij}}=1}}{\sum_{i=1}^{N}1\;l_{T_{i}=0}1\;l_{m_{\mathrm{ij}}=1}}\;\text{and}\;{\hat{q}}_{j}=\frac{\sum_{i=1}^{N}1\;l_{T_{i}=1}1\;l_{X_{\mathrm{ij}}=0}1\;l_{m_{\mathrm{ij}}=1}}{\sum_{i=1}^{N}1\;l_{T_{i}=1}1\;l_{m_{\mathrm{ij}}=1}},$$

where $m_{\mathrm{ij}}$ is equal to 1 if radiologist j observes mammography i and 0 otherwise.

### A Simulation Procedure for Synthetic Mammography Datasets

This paragraph details a simulation procedure to generate a dataset $X={\left\{{\left\{x_{\mathrm{ij}}\right\}}_{j},t_{i},n_{i}\right\}}_{i}$, see the example in Figure F7. $x_{\mathrm{ij}}$ s are filled with 0 and 1 corresponding to realizations of the variables $X_{\mathrm{ij}}$ for each of the n patients and each of the $n_{\mathrm{rad}}$ radiologists. This dataset must verify different points:

- $t_{i}$ is set equal to 0 for i∈[[1,84]] and to 1 for i∈[[85,148]]
- according to the equations (F9) and for any radiologist j, the ${\left(x_{\mathrm{ij}}\right)}_{i}$ s are such as follows:
  – there are exactly $84{\hat{p}}_{j}$ 1s and $84\left(1-{\hat{p}}_{j}\right)$ 0s in the 84 first coordinates of ${\left(x_{\mathrm{ij}}\right)}_{i}$
  – there are exactly $64{\hat{q}}_{j}$ 0s and $64\left(1-{\hat{q}}_{j}\right)$ 1s in the 64 last coordinates of ${\left(x_{\mathrm{ij}}\right)}_{i}$
- $n_{i}$ is set equal to $n_{\mathrm{rad}}$ except for radiologists of ids 1201, 7714 and 9007, as discussed in the following point.
- concerning the missing values discussed earlier:
  – there is 1 missing positive case for radiologist of id 1201 and $n_{i}$ is set to 109
  – there are 3 missing positive cases and 5 missing negative cases for radiologist of id 7714 and $n_{i}$ is set to 102
  – there is 1 missing negative case for radiologist of id 9007 and $n_{i}$ is set to 109.
- among the patients, some must be difficult to diagnose correctly.

For the last point we have chosen the following procedure for any radiologist j: treat $x_{j}^{\left(0\right)}≔{\left\{x_{\mathrm{ij}}\right\}}_{i\in \left[\;\left[1,84\right]\;\right]}$ and $x_{j}^{\left(1\right)}≔{\left\{x_{\mathrm{ij}}\right\}}_{i=\left[\;\left[85,148\right]\;\right]}$ in parallel. In $x_{j}^{\left(0\right)}$, there are exactly $84{\hat{p}}_{j}$ values equal to 1 and $84\left(1-{\hat{p}}_{j}\right)$ values equal to 0. In $x_{j}^{\left(1\right)}$, there are exactly $64{\hat{q}}_{j}$ values equal to 0 and $64\left(1-{\hat{q}}_{j}\right)$ values equal to 1. In both cases, we throw the desired positions of $84{\hat{p}}_{j}$ values among the vector [[1,84]], respectively the desired positions of $64{\hat{q}}_{j}$ values among the vector [[85,148]]. Each patient is weighted, based on weights $w_{i}$, corresponding to the difficulty of correctly diagnosing patient i (say $x_{\mathrm{ij}}=1$ *while* $t_{i}=0$ and $x_{\mathrm{ij}}=0$ *while* $t_{i}=1$) meaning that if $w_{k}>w_{i}$ then patient i is harder to diagnose correctly than patient k. Note, there are many possible datasets:

$$\prod_{j=1}^{n_{\mathrm{rad}}}C_{64}^{64{\hat{q}}_{j}}C_{84}^{84{\hat{p}}_{j}},$$

in the mean case ${\hat{p}}_{j}$ are all equal to $1/N\sum_{j=1}^{N}{\hat{p}}_{j}\approx 0.22$ and ${\hat{q}}_{j}$ are all equal to $1/N\sum_{j=1}^{N}{\hat{q}}_{j}\approx 0.13$ and the formula simplifies in ${\left(C_{64}^{32}C_{84}^{42}\right)}^{110}$, which is not reachable by common computers. So, testing all the possible datasets might be inappropriate. Although it is possible to derive the law of $x_{j}^{\left(0\right)}$ and $x_{j}^{\left(1\right)}$. Let us denote

$$U\left(\left[\;\left[\alpha ,\beta \right]\;\right],n_{0}\right)=\left\{\text{Draw of size}\;n_{0}\;\text{in}\;\left[\;\left[\alpha ,\beta \right]\;\right]\;\text{without replacement}\right\}.$$

Then $u\in U\left(\left[\;\left[1,84\right]\;\right],84{\hat{p}}_{j}\right)$ gives the indices of the non‐diseased women who are diagnosed as diseased by the radiologist. Denoting $u={\left\{u_{i}\right\}}_{i\in \left[\;\left[1,84{\hat{p}}_{j}\right]\;\right]}$:

$$\begin{array}{ll} & P\;\left({\left\{x_{\mathrm{ij}}^{\left(0\right)}\right\}}_{i\in u}=1,{\left\{x_{\mathrm{ij}}^{\left(0\right)}\right\}}_{i\in u^{c}}=0|{\hat{p}}_{j}\right)\\ & \;=\frac{\prod_{i\in \left[\;\left[1,84{\hat{p}}_{j}\right]\;\right]}w_{u_{i}}}{\prod_{i\in \left[\;\left[1,84{\hat{p}}_{j}\right]\;\right]}\left(\sum_{k\in u_{\left[\;\left[i,84{\hat{p}}_{j}\right]\;\right]}}w_{k}+\sum_{k\in u^{c}}w_{k}\right)}, \end{array}$$

where $u^{c}$ is the complementary of u in [[1,84]]. Equivalently, for any $v\in U\left(\left[\;\left[85,148\right]\;\right],64{\hat{q}}_{j}\right)$:

$$\begin{array}{ll} & P\;\left({\left\{x_{\mathrm{ij}}^{\left(1\right)}\right\}}_{i\in v}=0,{\left\{x_{\mathrm{ij}}^{\left(1\right)}\right\}}_{i\in v^{c}}=1|{\hat{q}}_{j}\right)\\ & \;=\frac{\prod_{i\in \left[\;\left[85,64{\hat{q}}_{j}\right]\;\right]}w_{v_{i}}}{\sum_{i\in \left[\;\left[85,64{\hat{q}}_{j}\right]\;\right]}\left(\sum_{k\in v_{\left[\;\left[i,64{\hat{q}}_{j}\right]\;\right]}}w_{k}+\sum_{k\in v^{c}}w_{k}\right)}, \end{array}$$

where $v^{c}$ is the complementary of v in [[85,148]]. These probabilities were calculated by considering the order, which is the solution chosen by the function sample of the software **R** [24].
